# Development of a base editor for protein evolution via *in situ* mutation *in vivo*

**DOI:** 10.1093/nar/gkab673

**Published:** 2021-08-14

**Authors:** Wenliang Hao, Wenjing Cui, Zhongyi Cheng, Laichuang Han, Feiya Suo, Zhongmei Liu, Li Zhou, Zhemin Zhou

**Affiliations:** The Key Laboratory of Industrial Biotechnology (Ministry of Education), School of Biotechnology, Jiangnan University, 1800 Lihu Avenue, Wuxi 214122, China; The Key Laboratory of Industrial Biotechnology (Ministry of Education), School of Biotechnology, Jiangnan University, 1800 Lihu Avenue, Wuxi 214122, China; The Key Laboratory of Industrial Biotechnology (Ministry of Education), School of Biotechnology, Jiangnan University, 1800 Lihu Avenue, Wuxi 214122, China; The Key Laboratory of Industrial Biotechnology (Ministry of Education), School of Biotechnology, Jiangnan University, 1800 Lihu Avenue, Wuxi 214122, China; The Key Laboratory of Industrial Biotechnology (Ministry of Education), School of Biotechnology, Jiangnan University, 1800 Lihu Avenue, Wuxi 214122, China; The Key Laboratory of Industrial Biotechnology (Ministry of Education), School of Biotechnology, Jiangnan University, 1800 Lihu Avenue, Wuxi 214122, China; The Key Laboratory of Industrial Biotechnology (Ministry of Education), School of Biotechnology, Jiangnan University, 1800 Lihu Avenue, Wuxi 214122, China; The Key Laboratory of Industrial Biotechnology (Ministry of Education), School of Biotechnology, Jiangnan University, 1800 Lihu Avenue, Wuxi 214122, China

## Abstract

Protein evolution has significantly enhanced the development of life science. However, it is difficult to achieve *in vitro* evolution of some special proteins because of difficulties with heterologous expression, purification, and function detection. To achieve protein evolution via *in situ* mutation *in vivo*, we developed a base editor by fusing nCas with a cytidine deaminase in *Bacillus subtilis* through genome integration. The base editor introduced a cytidine-to-thymidine mutation of approximately 100% across a 5 nt editable window, which was much higher than those of other base editors. The editable window was expanded to 8 nt by extending the length of sgRNA, and conversion efficiency could be regulated by changing culture conditions, which was suitable for constructing a mutant protein library efficiently *in vivo*. As proof-of-concept, the Sec-translocase complex and bacitracin-resistance-related protein BceB were successfully evolved *in vivo* using the base editor. A Sec mutant with 3.6-fold translocation efficiency and the BceB mutants with different sensitivity to bacitracin were obtained. As the construction of the base editor does not rely on any additional or host-dependent factors, such base editors (BEs) may be readily constructed and applicable to a wide range of bacteria for protein evolution via *in situ* mutation.

## Graphical Abstract

**Graphical Abstract:**
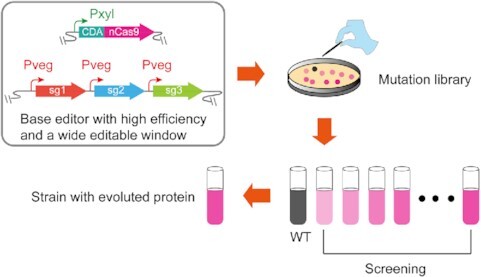
The diagram for the construction of the base editor and its working process for protein evolution.

## INTRODUCTION

Protein evolution is one of the most important research topics in protein engineering, significantly enhancing the development of life sciences. It usually requires multisite mutations (1–3). For example, protein directed evolution requires multiple mutants by error-prone PCR, or chemical or physical mutagenesis, to construct a mutant library. Then, the desired mutants need to be screened and isolated from the library. Though error-prone PCR can perform protein evolution *in vitro*, this framework is difficult to use for some special proteins, such as membrane, toxic, labile proteins, and protein complex, because of the difficulty with their heterologous expression, purification and function detection. Moreover, protein complexes exhibit functions in the entirety of a system (4), with the function of each protein being undetectable *in vitro*. Therefore, a framework is required for protein evolution through *in situ* mutation *in vivo*. The rapid development of genome editors makes the framework construction possible.

A genome editor based on CRISPR-Cas system has been widely used to engineer gene sequences (5–9). Widely used Cas proteins are Cas9 (10) and Cas12a (11). For these systems, the Cas proteins with specific gRNA are first guided to a target loci by recognizing a protospacer adjacent motif (PAM), after which the genomic DNA is cut at the locus to produce a double-strand break (DSB) (12). The cells repair the DSB through heterologous homology-directed repair (HDR) or endogenous non-homologous end joining (NHEJ) to survive (13–15). Based on the CRISPR-Cas system, a new genome editor named base editor (BE) was recently developed. The BE was designed by fusing Cas9 variants with cytidine deaminase (CDA) or adenine deaminase (ADA) to edit genes at the single nucleotide level (16–18). Cas9 variants, which mainly include Cas9 nickase (D10A, usually denoted nCas9) and catalytically inactive Cas9 (D10A/H840A, usually denoted dCas9), fuse with deaminases to induce C:G to T:A or A:T to G:C conversion (19). Numerous tools derived from BEs have been successfully applied in gene insertions, gene deletions, and point mutations in various bacterial (20–24), mammalian (25–27) and plant cells (28–30). Due to the diverse genome environments in diverse cells and host-dependent factors of some tools, one tool is usually applied for one kind of cell. In addition, the tools also displayed significantly different conversion efficiency even in one kind of cell because of the different expression version and construction strategy (31,32). No matter how to use these tools, a BE with high stability and high conversion efficiency is expected.

Here, we developed a highly efficient BE named CRISPR-CDA-nCas9-UGI in *Bacillus subtilis* employing nCas9 and a cytosine deaminase through genome integration. The BE induced a cytidine-to-thymidine mutation of approximately 100% in an editable window, much higher than that of other BEs. The editable window and conversion efficiency could be easily adjusted, which was suitable for constructing a mutant protein library efficiently. As proof-of-concept, the Sec-translocase complex, a membrane protein complex involved in protein transportation in *B. subtilis*, was successfully evolved *in vivo*. Based on the evolved Sec-translocase complex, a host cell with a 3.6-fold translocation efficiency was obtained. Additionally, a membrane protein, bacitracin resistance related protein (BceB) in *B. subtilis* was also successfully evolved *in vivo*, and two mutants with high and low resistance capacity to bacitracin were isolated, respectively. As the construction of the base editor does not rely on any additional or host-dependent factors, such BEs may be readily constructed and applicable to a wide range of bacteria for protein evolution via *in situ* mutation, especially applicable to membrane, toxic, labile proteins, and protein complex.

## MATERIALS AND METHODS

### Bacterial strains, media and growth conditions

The strains used in this study are described in Supplementary Table S1. The *Escherichia coli* JM109 strains (General Biosystems, China) were used as the host for plasmid propagation. *E. coli* strains were grown aerobically at 37°C in Luria–Bertani (LB) liquid medium (10 g/l tryptone, 5 g/l yeast extract, 10 g/l NaCl, pH 7.0) or on LB-agar plates supplemented with ampicillin (100 μg/ml) or spectinomycin (50 μg/ml) when necessary. *B*. *subtilis* 168 strains were cultivated in LB liquid medium or LB solid medium supplemented with spectinomycin (50 μg/ml), chloramphenicol (5 μg/ml), kanamycin (50 μg/ml), and tetracycline (15 μg/ml). For antimicrobial assays, *B*. *subtilis* 168 strain and mutants were cultivated in LB-agar plates supplemented with 0.2 mg/ml or 2 mg/ml bacitracin.

### DNA manipulation

The primers used for gene cloning are listed in Supplementary Table S2. The plasmids used in this study are listed in Supplementary Table S3. PCR and DNA digestion were performed according to the manufacturer's instructions. Two or more DNA fragments were ligated using the Seamless Cloning Kit (purchased from General Biosystems (Anhui) Co., Ltd.). *E. coli* JM109 was transformed by standard chemical transformation. *B. subtilis* was transformed by the modified Spizizen's transformation method (33). A marker-free genome editing approach was used to perform gene overexpression in *B. subtilis* as previously reported (34).

### Design of sgRNAs

The general sgRNAs were designed based on a previous study (35) via online software (http://chopchop.cbu.uib.no/) (36). The 3′-terminal extended sgRNAs for the target region (N20) were designed by gradually adding the corresponding base. The artificial stem loop was designed via RNAfold (37). The relevant sgRNA sequences are listed in Supplementary Table S4.

### Plasmids construction

The targeting plasmid pHY300PLK (kindly provided as a gift from Prof. Guimin Zhang from Hubei University) was used as the backbone to construct the sgRNA-expression vectors (38). The plasmid pHY-ECBE harbouring *sigE*-targeting sgRNAs was constructed by reverse PCR (rPCR) using prime pHY-ECBE-F/pHY-ECBE-R.

To integrate the dCas9 or nCas9 and cytosine deaminase (CDA) fusion into the genome of *B. subtilis*, plasmids pAX-nCas9 and pAX-dCas9 were constructed by the insertion of nCas9 and dCas9 downstream of the xylose-inducible promoter P_xylA_ on the integration plasmid pAX01, respectively. Accordingly, pAX-nCas9, pAX-dCas9, and CDA (synthesized by GENEWIZ, Inc. SuZhou, China) were amplified using primers n-CDA-b-F/n-CDA-b-R (used by both fragments) and n-CDA-F/n-CDA-R. The PCR product of the CDA fragment was then assembled to each of the two linearized plasmids using the Gibson assembly method, yielding two plasmids, pAX-CDA-nCas9 and pAX-CDA-dCas9, harbouring a CDA-dCas9 fusion with a 6aa protein linker between them.

To improve the mutation rate, uracil DNA glycosylase inhibitor (UGI) from bacteriophage PBS2 (39) was introduced into pAX-CDA-nCas9, placed at the C-terminus of the CDA-6aa-nCas9 fusion using Gibson assembly, yielding pAX-CDA-nCas9-UGI.

The plasmid pAD123 was used as a template to construct sgRNA targeting *secY* for single editing. Plasmids targeting different positions of *secY* were constructed by reverse PCR using the primers pHY-T1-F/pHY-T1-R, pHY-T2-F/pHY-T2-R and pHY-T3-F/pHY-T3-R, yielding pAD-secYT1, pAD-secYT2 and pAD-secYT3, respectively. Then, sgRNA targeting secY(T1), secY(T2), and secY(T3) were separately cloned into the integration vector pDGT-GFP (38) using primers pDG-T1-F/pDG-T1-R, pDG-T2-F/pDG-T2-R and pDG-T3-F/pDG-T3-R, producing pDG-T1, pDG-T2 and pDG-T3, respectively.

To construct a multiplex base editing system for the modification of the SecYEG complex, plasmid pAD123 (35) was used as the donor vector for the expression of sgRNA driven by a strong constitutive promoter P_veg_. Using the pAD123, the plasmids pAD-T0, pAD-T3, pAD-secE, and pAD-secG that harboured sgRNAs targeting *secY* (T0), *secY* (T3), *secE* (ET) and *secG* (GT), respectively, were generated by reverse PCR using primers secYT0-F/secYT0-R, secYT3-F/secYT3-R, secEET-F/secEET-R and secGGT-F/secGGT-R, respectively.

The pDGT-GFP was used as the backbone to construct plasmids harbouring sgRNAs for multiplex base editing. To generate dual-editing plasmids targeting *secY* and *secE*, pDG-T0ET harbouring sgRNAs targeting secY(T0) and secE(ET) sites were constructed using primers secY-Go-dual-F/secY-Go-dual-R, secE-Go-dual-F/secE-Go-dual-R, and secYE-Go-b-F/secYE-Go-b-R. Similarly, pDG-T3ET harbouring sgRNAs secY(T3) and secE(ET) were constructed to target secY(T3) and secE(ET) sites using the same primers as above.

Triple-editing plasmids pDG-T0ETGT and pDG-T3ETGT were constructed using pDGT-GFP-Amp_m_ as the backbone. The backbone was first linearized by PCR using primers secYE-Go-b-F/secYE-Go-b-R. The three fragments of sgRNAs harbouring secY(T0), secE(ET), and secG(GT) were separately amplified using primers secY-Go-dual-F/secY-Go-dual-R, secE-Go-dual-F/secE-Go-tri-R, and secG-Go-tri-F/secE-Go-dual-R, respectively. They were then ligated with the linearized backbone by Golden Gate assembly, producing pDG-T0ETGT. Similarly, pDG-T3ETGT harbouring sgRNAs secY(T3), secE(ET) and secG(GT) was constructed using the same primers as above.

The construction of dual-editing plasmids (pDG-B3-6, pDG-B4-5 and pDG-B1-8) and triple editing plasmids (pDG-B3-6-9, pDG-B4-5-6 and pDG-B1-8-10) can refer to the construction of pDG-T0ET and pDG-T0ETGT mentioned above.

### Plasmid curing

Colonies confirmed by Sanger sequencing were inoculated into LB media containing 0.005% sodium dodecyl sulfate (SDS) without antibiotics, and incubated at 37°C, 200 rpm for approximately 20 h for plasmid curing (40). Accordingly, the culture was diluted and spread on LB plates without antibiotics. Colonies were carefully picked up and dotted at the same positions on two LB plates with and without antibiotics. Antibiotic-sensitive colonies were picked and propagated in 5 ml LB medium. Then, plasmid-free mutants were further confirmed through sequencing.

### Construction of a single-editing system

To construct a single-editing system, integration vector pAX-CDA-nCas9-UGI (or pAX-CDA-nCas9) harbouring fusion protein CDA-nCas9-UGI (or CDA-nCas9) transformed into the genome of *B. subtilis* at the *lacA* site. For the editing of *sigE*, plasmid harbouring single sgRNA was introduced into the CDA-nCas9-integrated host. For editing *secY*, three sgRNAs randomly targeting different positions on *secY* were designed. These sgRNAs harbored identical crRNA scaffold (35) with 20-nt targeting sequence downstream. These sgRNAs were integrated into the *amyE* site of CDA-nCas9-UGI-integrated cells, respectively. After the function of each mutant was determined, the same system was reconstructed into a plasmid in order to eliminate the system for construction of chassis cells. For *bceB* mutagenesis experiment, the same method was employed.

### Construction of dual- and triple-editing systems

To construct dual-editing and triple-editing systems for the Sec mutagenesis experiments, sgRNAs with 20 nt targeting secY (T3), secE (ET) and secG (GT) were differentially combined, generating T3/ET, ET/GT and T3/ET/GT. These different sgRNA combinations were integrated into the *amyE* site of CDA-nCas9-UGI-integrated cells, respectively. After the function of each mutant was determined, specific sgRNA combinations were constructed into a plasmid (e.g. pHT-ETGT) in order to obtain powerful chassis cells. The construction of multiplex gene editing system targeting *bceB* was similar to Sec mutagenesis experiments.

### DNA sequencing at the single-clone level and population level

Two methods of sequencing, single-clone and population-level sequencing, were used to analyse the editing efficiency of the BE. The colonies were randomly selected from the LB-agar plates after induction by xylose regarding the single-clone sequencing. Colony PCR was performed on the selected colonies and the PCR products were sequenced to verify the C-to-T conversion. The colonies were cultured for approximately 10 h in a test tube containing 1% of xylose. Then, the induced culture was used as templates to amplify the position of the expected mutation by using customized primer pairs. The PCR products were used for population sequencing and the raw data were analysed by online software (https://moriaritylab.shinyapps.io/editr_v10/) (41).

### Detection of GFP by multi-functional microplate reader

To characterize the change of secretory performance, we employed the medium and intracellular fluorescence intensity (FI) ratio as the evaluation criteria to characterize the change in secretory performance. *B. subtilis* with modified and unmodified Sec pathways were transformed with pB-P_srfA_-WapA-GFP harbouring a Sec-dependent signal peptide WapA preceding the reporter gene GFP. The recombinant strains were cultured overnight and collected for further analysis. Cells and media were separated via centrifugation at 12,000 rpm for 10 min. The supernatant was carefully absorbed and the cells were resuspended with the corresponding volume of deionized water. The supernatant and cell suspension (each 200 μl) were transferred into 96-well black-walled plates and analysed using a PerkinElmer EnSpire^®^ 2300 Multimode Plate Reader (excitation at 495 nm and emission at 525 nm).

### Agar diffusion growth inhibition assay

The growth inhibition activity of bacitracin was determined against mutant *B. subtilis* 168 (B9 and B10). Wild type *B. subtilis* 168 strains were used as controls. Luria–Bertani agar (add 0.75% agar and 1% Tween-20) was cooled to 42 °C and seed with 1% overnight culture (approximately 10^8^–10^9^ CFU ml^–1^) of the indicator strains (wild type and mutant *B. subtilis* 168). After agar solidification in a Petri dish, samples were applied to a small well created on the agar plates. Assay samples were typically diluted to final concentrations of 0.2 and 2 mg/ml. Plates were incubated at 4°C for 4 h for pre-diffusion, then transfer the plates to 30°C for 14–16 h. The resistance of different mutants to bacitracin was qualitatively determined by the size of a zone of growth inhibition.

### Next-generation sequencing and off-target analysis

The culture of the base-edited cells was prepared. Approximately, 10^9^ cells were used for extraction of genomic DNAs. NEBNext Ultra DNA Library Prep Kit was used to convert the amplicon into indexed libraries for Next-generation sequencing (NGS) on the Illumina platform. Library construction and sequencing were performed by GENEWIZ (Suzhou, China). Approximately 20 000 000–30 000 000 reads per sample were analyzed. Base-substitution frequencies were calculated by dividing base-substitution reads by total reads. For NGS off-target analysis, off-target sites of selected target loci were analyzed by Cas-OFFinder (42). All similar sequences of selected target loci were chosen as predicted off-target sites (Supplementary Table S6).

### Statistical analysis

Statistical significance between two editing system was assessed by comparing the mean values (±S.D.) using Student's *t* test. *P* < 0.01 was considered significant (**P < 0.01).

## RESULTS

### Construction of BEs via CRISPR-Cas9

**Figure 1.**
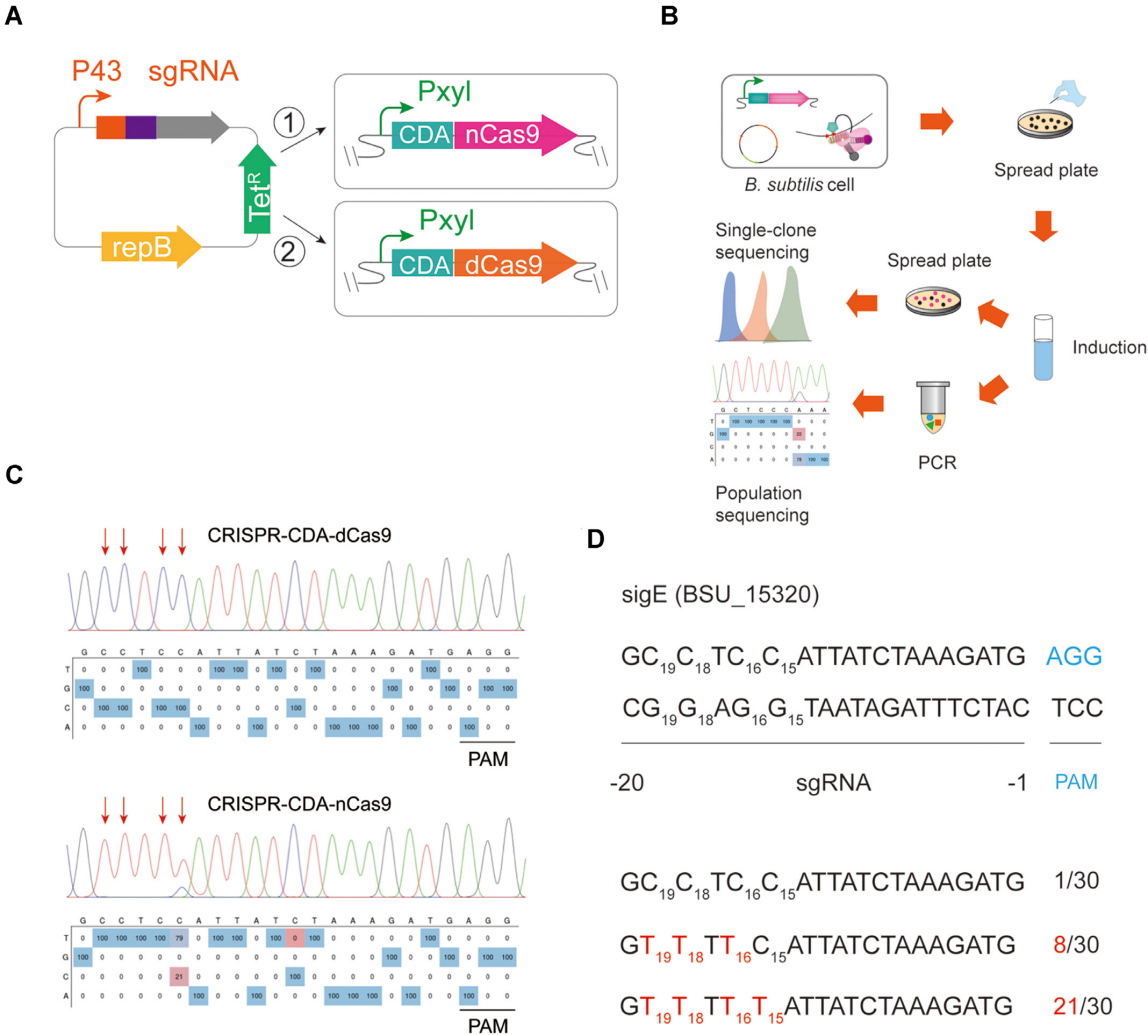
Construction and characterization of CRISPR-CDA-Cas9-mediated base editor system in *B. subtilis*. (**A**) Design and construction of CRISPR-CDA-Cas9 mediating base editors. The fusion protein comprising nCas9/dCas9 and CDA under the control of xylose promoter P_xylA_ was integrated into the *lacA* locus in *B. subtilis*. The repressor gene *xylR* was constitutively expressed in the expression cassette. A strong constitutive promoter P43 controlled the designed sgRNA. (**B**) Workflow for gene editing identification and verification at the single-clone level and population level in *B. subtilis*. (**C**) The editing efficiency of CRISPR-CDA-dCas9 and CRISPR-CDA-nCas9 at *sigE* site. (**D**) Base conversion targeting *sigE* at the single-clone level. Thirty colonies were selected and subjected to gene sequencing and the conversion efficiency was calculated. The PAM sequence is blue, whereas the modified bases are red.

To obtain a genome editor suitable for multisite mutations with a high conversion efficiency, two versions of a BE based on the CRISPR-Cas9 system were constructed in *B. subtilis* 168 (Figure 1A). The CDA from *Petromyzon marinus* was fused at the N-terminal of dCas9 and nCas9 (CRISPR-CDA-dCas9 and CRISPR-CDA-nCas9). The fused CRISPR-CDA-dCas9 and CRISPR-CDA-nCas9 genes were specifically integrated into the *lacA* locus of the *B. subtilis* 168 genome through the integration vector pAX01. One of the sigma factors, sigE in *B. subtilis*, was selected as the target protein for editing, and the plasmid (pHY-ECBE) used for sgRNA expression targeting *sigE* was constructed using a strong promoter, P43 of *B. subtilis* (Figure 1A). The pHY-ECBE was transformed into *B. subtilis* 168 harbouring CRISPR-CDA-dCas9 and CRISPR-CDA-nCas9 genes, yielding BS1-sigE and BS2-sigE, respectively.

### Characterization of the BEs

The conversion efficiency of the two systems was tested after incubation and induction (Figure 1B). For BS1-sigE, the culture was absorbed to perform PCR using customized primer pairs and sequenced to detect the mutation efficiency at the target loci. However, no base conversion was observed in an editable window (Figure 1C). Conversely, high conversion efficiency with a wide editing window was detected in BS2-sigE (Figure 1C). The population sequencing results showed that base conversion occurred at four sites; the conversion efficiency in C15, C16, C18, and C19 was 79%, 100%, 100% and 100%, respectively (Figure 1C). These results demonstrated that the BE of CRISPR-CDA-nCas9 has the potential for base editing in *B. subtilis*.

To certify the reliability of the CRISPR-CDA-nCas9 on base editing, the conversion efficiency was verified at the single-clone level. Theoretically, the custom sgRNA carries the CDA-nCas9 fusion to the specific location, where deamination occurs in an editable window. The culture of BS2-sigE was diluted and spread onto a plate after incubation and induction; 30 colonies were randomly selected and sequenced at the target loci (Figure 1B). As shown in Figure 1D, the four cytosine bases of C15, C16, C18 and C19 were successfully converted to T with conversion efficiencies of 21/30, 29/30, 29/30 and 29/30, respectively. These results demonstrated that CRISPR-CDA-nCas9 exhibited a higher conversion efficiency with an editable window of 5 nt.

### Effect of inducer concentration on conversion efficiency

**Figure 2.**
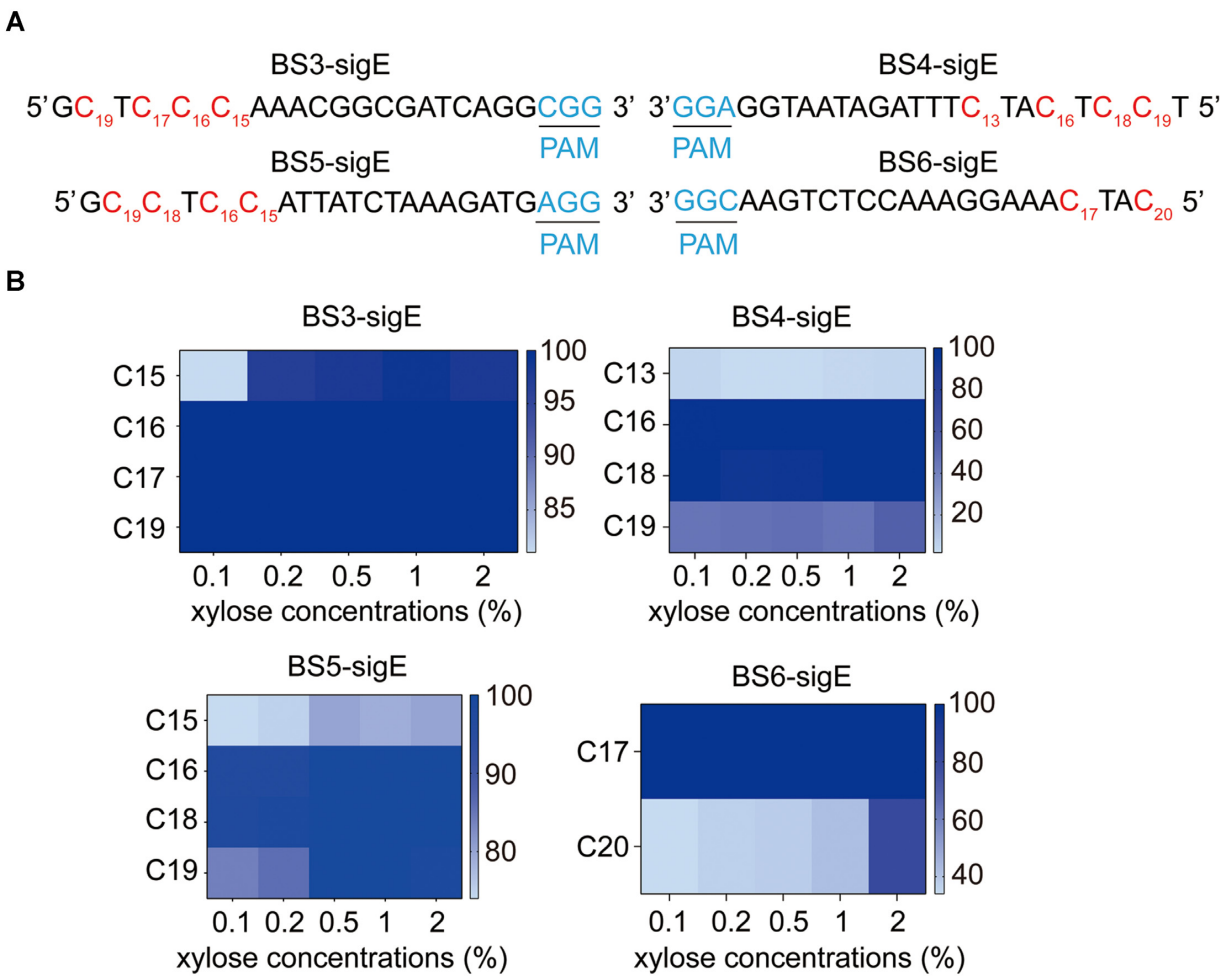
Conversion efficiency of BS3-sigE, BS4-sigE, BS5-sigE and BS6-sigE with different inducer concentrations. (**A**) Four designed sgRNAs targeting four *sigE* locus sites in the four strains. Each of the PAM motifs is shown in blue, the modified bases are shown in red. (**B**) Conversion efficiency of each strain with different inducer concentrations.

Protein expression level may affect the conversion efficiency of CRISPR-CDA-nCas9. Therefore, the relationship between the concentration of inducer and conversion efficiency was tested. Four sgRNAs targeting different loci of *sigE* were designed (Figure 2A), and plasmids containing the four sgRNAs were transformed into *B. subtilis* harbouring the CRISPR-CDA-nCas9 system, yielding BS3-sigE, BS4-sigE, BS5-sigE and BS6-sigE. These transformants were induced by xylose with final concentrations of 0.1%, 0.2%, 0.5%, 1% and 2%. Population sequencing showed that several sites with high conversion efficiency were not affected by the high concentration of xylose. The conversion efficiency in other sites increased as the xylose concentration increased, and usually, 1% of xylose was enough for a high conversion efficiency (Figure 2B). Additionally, the conversion efficiency of C15 in BS3-sigE and C20 in BS6-sigE reached 90% and 80%, respectively (Figure 2B), indicating that the BE had an editable window of at least a 6 nt. Moreover, the middle sites of the editable window exhibited higher conversion efficiency, whereas the conversion efficiency of the sites in the two flanks of the editable window was lower (Figure 2B). These variations might be attributed to the genetic environment of the genome (43).

### Improving the conversion efficiency and expanding the editable window of CRISPR-CDA-nCas9

**Figure 3.**
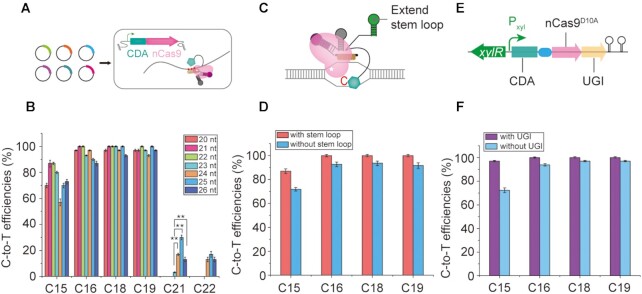
Improving the conversion efficiency and expanding the editable window of CRISPR-CDA-nCas9. (**A**) Design and construction of CRISPR-CDA-Cas9 mediating base editors with different sgRNA. (**B**) Effects of sgRNA length on conversion efficiency and editable window. The asterisks indicate significant editing based on a comparison between the experimental group and control group (**P <0.01, Student's *t*-test). (**C**) Design of an artificial stem loop fused with the 3′ terminus of sgRNA. (**D**) Effects of a fused artificial stem loop on conversion efficiency. (**E**) Design of UGI fused with the C-terminus of CDA-nCas9. (**F**) Effects of UGI fused with the C-terminus of CDA-nCas9 on conversion efficiency. Bars represent the average editing efficiency and error bars represent the S.D. of three independent biological replicates.

It has been reported that extending the sgRNA targeting region enables the expansion of the editing window (21,31). To further expand the editable window of CRISPR-CDA-nCas9, sgRNAs consisting of a 21, 22, 23, 24, 25 and 26 nt targeting *sigE* were designed (Figure 3A). Compared with a conventional 20 nt sgRNA, the conversion efficiency increased on prolonging sgRNAs, with the highest conversion efficiency occurring when the 21 and 22 nt sgRNAs were used (Figure 3B). In addition, the C-to-T conversions at C21 and C22 were not observed using 21- and 22-nt sgRNA. Interestingly, sgRNAs in the length of 23 nt, 24 nt, 25 nt, and 26 nt expanded the editable window to 8 nt (Figure 3B). Especially, extending sgRNA from 23 nt to 24 nt, 25 nt and 26 nt significantly increased the conversion efficiency at C21 position (**P < 0.01, Student's *t*-test). These results suggest that extending the length of sgRNA of the CRISPR-CDA-nCas9 presents a trend for expanding the editable window and improves conversion efficiency.

Moreover, a previous report has shown that an artificial stem loop fused with the 3′ terminal of sgRNA could recruit CDA-fused protein for base editing (44). According to this result, an artificial stem loop was fused to the 3′ terminal of a 20-nt sgRNA (Figure 3C). Sequencing results showed that the conversion efficiency of C15, C16, C18 and C19 was 87%, 100%, 100% and 100%, respectively (Figure 3D). Compared with a 20-nt sgRNA without an artificial stem loop (70%, 100%, 100% and 100%, respectively), these results indicate that the modification of sgRNA at the 3′ terminus improved the conversion efficiency of CRISPR-CDA-nCas9.

UGI can inhibit the reverse mutation of T to C (31). To avoid the reverse mutation from reducing the conversion efficiency of CRISPR-CDA-nCas9, UGI was fused with the C-terminus of CDA-nCas9, resulting in CRISPR-CDA-nCas9-UGI (Figure 3E). The conversion efficiency of CRISPR-CDA-nCas9-UGI was detected. As shown in Figure 3F, the conversion efficiency of C15, C16, C18, and C19 reached 97%, 100%, 100% and 100%, respectively, indicating that upgrading the editor to CRISPR-CDA-nCas9-UGI enhanced the conversion efficiency.

### Conversion efficiency for multi-genes of CRISPR-CDA-nCas9-UGI

The CRISPR-CDA-nCas9-UGI achieved multisite mutations in one gene. To test whether it could achieve base editing for multi-genes, the Sec-translocase complex from *B. subtilis* was chosen as target proteins. These were SecY, SecE and SecG on the outer-membrane (45). A series sgRNA targeting double genes of *secY* and *secE* and a series sgRNA targeting triple genes of *secY*, *secE* and *secG* were designed (Supplementary Figure S1). To maintain the stable expression of sgRNAs, the two sgRNAs were integrated on the *amyE* locus of the *B. subtilis* genome, harbouring CRISPR-CDA-nCas9-UGI. After incubation and induction by 1% xylose, PCR was performed using the mixed culture as a template. The sequencing results for the double gene editing showed that the conversion efficiencies of C15 and C16 towards *secY* and that of C18 towards *secE* were 8%, 1%, and 96%, respectively (Supplementary Figure S2). For the triple gene editing, no C-to-T conversions occurred at either C15 and C16 towards *secY*, and the conversion efficiency of C18 for *secE* and C20, C17 and C16 for secG was 100%, 62%, 96% and 97%, respectively (Supplementary Figure S2). Although the conversion efficiency was lowered by a certain extent, these results suggest that CRISPR-CDA-nCas9-UGI could be applied for base editing of multi-genes at different sites.

**Figure 4.**
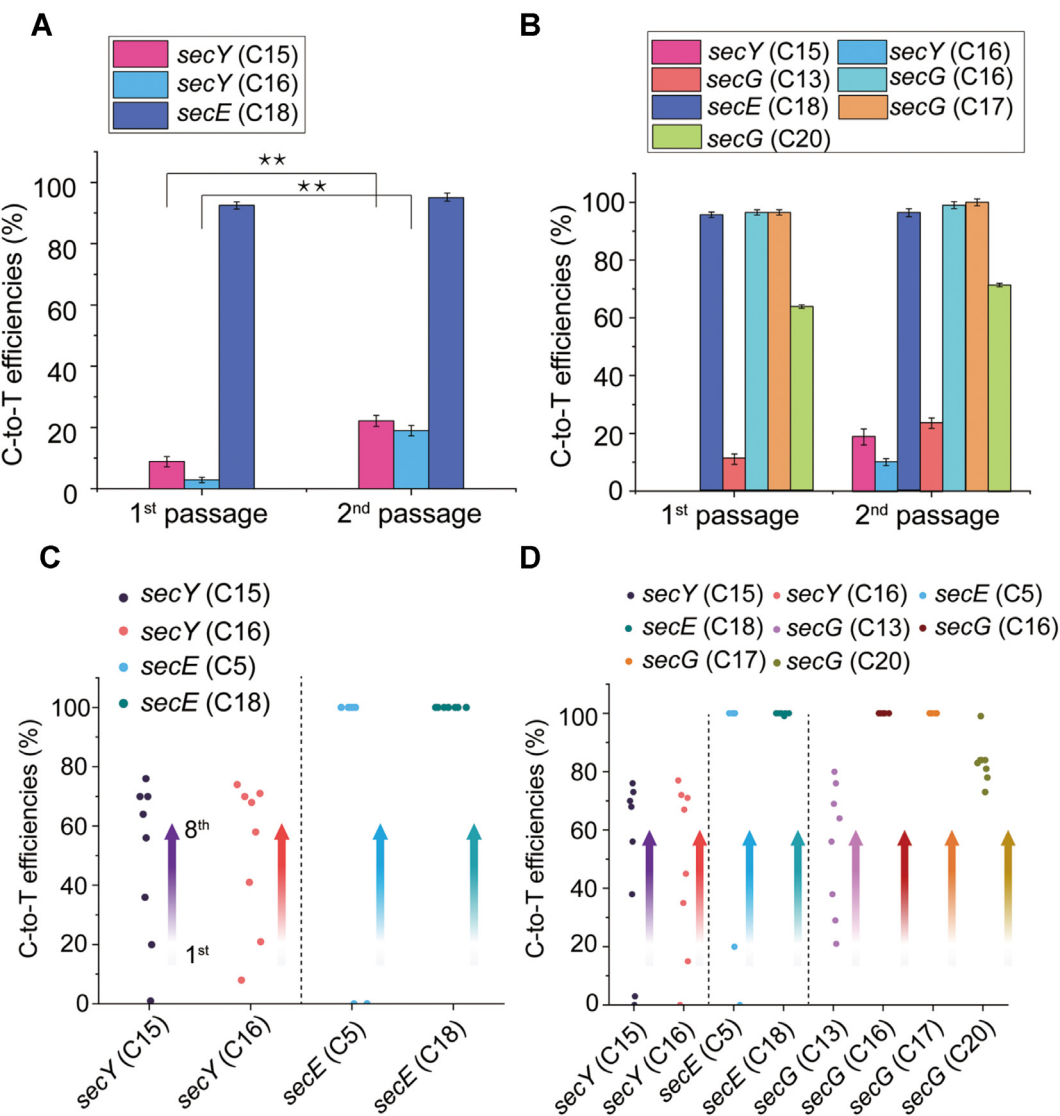
Conversion efficiency on multi-genes of CRISPR-CDA-nCas9-UGI. (**A**) Conversion efficiency of double genes (*secY* and *secE*) editing. (**B**) Conversion efficiency of triple genes (*secY*, *secE*, and *secG*) editing. The conversion efficiency of the first and second passage for the double and triple genes was compared. The asterisks indicate significant editing based on a comparison between the experimental group and control group (**P <0.01, Student's *t*-test). Conversion efficiency increased with the multiple iterations for the (**C**) double and (**D**) triple genes. The direction of the arrow indicates the increase with the passage. Bars represent the average editing efficiency and error bars represent the S.D. of three independent biological replicates.

Iteration was conducted to improve the conversion efficiency of multiplex editing. The sequencing results showed that the conversion efficiency of the second passage was significantly higher than that of the first passage (Figure 4A), especially for the low editing sites, C15 and C16 (**P < 0.01, Student's *t*-test), indicating that iteration was a promising way to enhance conversion efficiency for multiplex base editing. Therefore, multiple iterations (up to eight passages) were performed to further increase the conversion efficiency at each editable site of *secY*, *secE* and *secG*. As shown in Figure 4B and Supplementary Figure S3, the conversion efficiency of both double and triple gene editing significantly increased.

### Sec-translocase evolution of *B. subtilis* by CRISPR-CDA-nCas9-UGI

**Figure 5.**
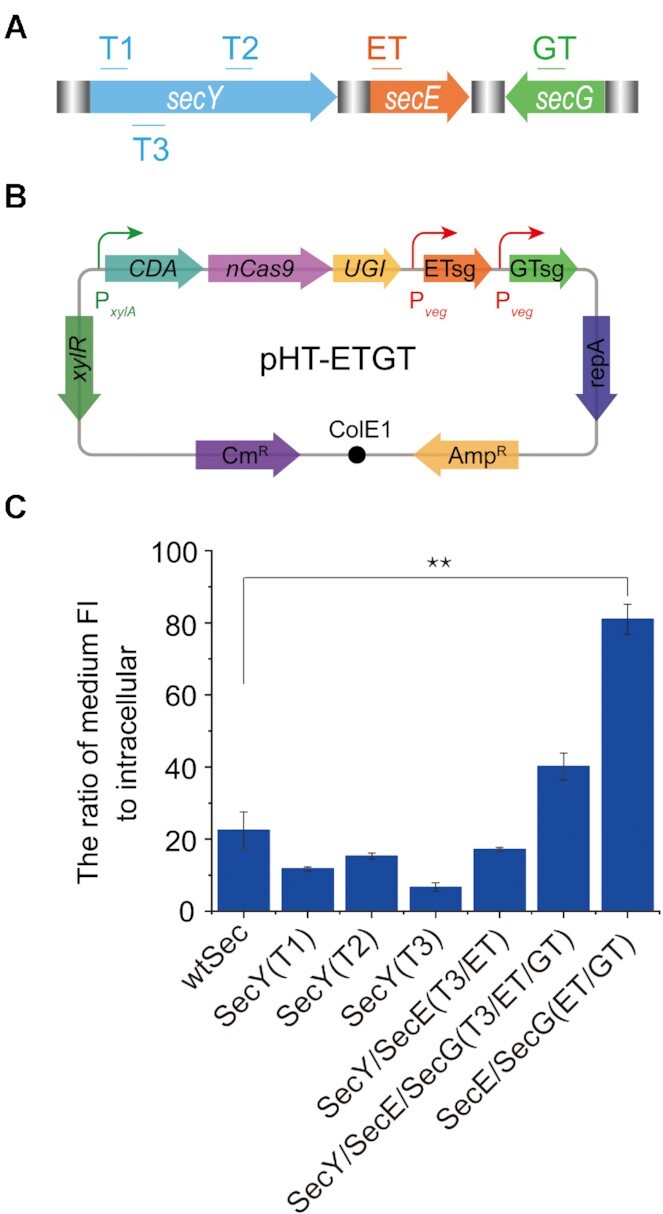
Evolution of the Sec-translocase in *B. subtilis* by CRISPR-CDA-nCas9-UGI. (**A**) Selection of sgRNA targeting specific sites in *secY*, *secE* and *secG*. Three sgRNAs (T1, T2 and T3) targeting *SecY*, a series sgRNA (T3/ET) targeting double genes of *secY* and *secE*, and a series sgRNA (T3/ET/GT) targeting triple genes of *secY*, *secE* and *secG* were designed. (**B**) The BE constructed in a plasmid used for manufacture of the mutant SecE(V36I)/SecG(A62T/V63I). (**C**) Comparison of the transportation ability among the wild type Sec translocase and its mutants. Bars represent the average transportation efficiency and error bars represent the S.D. of three independent biological replicates. The asterisks indicate significant editing based on a comparison between the experimental group and control group (**P <0.01, Student's *t*-test).

To test whether the CRISPR-CDA-nCas9-UGI was suitable for protein evolution by *in situ* mutation *in vivo*, the Sec*-*translocase complex in *B. subtilis* was selected as the target protein. As SecY serves as the export tunnel among the translocase complex, which directly influences the transporting function (46), three sgRNAs (T1, T2 and T3) targeting *secY* were randomly designed (Figure 5A). Furthermore, a series sgRNA (T3/ET) targeting double genes, *secY* and *secE*, and a series sgRNA (T3/ET/GT) targeting triple genes, *secY*, *secE* and *secG*, were designed for double and triple gene editing (Figure 5A). The designed sgRNAs were integrated on the *amyE* locus of the *B. subtilis* genome harbouring CRISPR-CDA-nCas9-UGI and yielded SecY(T1), SecY(T2), SecY(T3), SecY/SecE(T3/ET) and SecY/SecE/SecG(T3/ET/GT), respectively.

The five strains were incubated under different concentrations of xylose (0.1–1%) to obtain as many mutants as possible for constructing a mutant library. Each strain was randomly isolated under the induction of different concentrations of xylose from the mutant library and sequenced (Supplementary Figure S4 and Supplementary Table S5). GFP was used to test the performance of protein transportation. Plasmid pB-P_srfA_-WapA-GFP harbouring a Sec-dependent signal peptide WapA preceding the reporter gene GFP was transformed into each mutant. The supernatant and cells were separately collected and the fluorescence ratio of the supernatant to whole cell (FI^sup^/FI^cell^) was calculated after incubation. Compared with the wild type, although the transportation abilities of four mutants decreased, one of the mutants, SecY/SecE/SecG(T3/ET/GT), exhibited a 1.8-fold increase in transportation ability (Figure 5C). These results showed that the evolution of the Sec*-*translocase complex in *B. subtilis* achieved *in vivo* mutants by the BE of CRISPR-CDA-nCas9-UGI.

The mutant of SecY/SecE/SecG(T3/ET/GT) which included two mutant sites in SecY(L55F/l56F), one mutant site in SecE (V36I), and two mutant sites in SecG (A62T/V63I) (Table S5), exhibited a 1.8-fold increase in transportation ability, while the mutant of SecY(T3) containing the two same mutant sites in SecY(L55F/l56F) showed sharp reduce in transportation ability. These results indicated that transportation ability may be increased by back mutation of L55F/l56F in SecY. Therefore, a BE constructed in a plasmid containing CDA-nCas9-UGI genes and a series sgRNA (ET/GT) targeting genes of *secE* and *secG* (Figure 5B), were used for mutant manufacture of SecE(V36I)/SecG(A62T/V63I). After confirmation by sequencing, the plasmid used for base editing was dispelled and the transportation ability of the mutant was detected. As shown in Figure 5C, the mutant of SecE(V36I)/SecG(A62T/V63I) exhibited a 3.6-fold increase in transportation ability, which was significantly higher than that of the wild-type (**P < 0.01, Student's *t*-test). These results demonstrated that the protein evolution information caused by CRISPR-CDA-nCas9-UGI could provide guidelines for powerful host cell obtaining through protein engineering.

### Evolution of Bacitracin-resistant protein (BceB) in *B. subtilis* by CRISPR-CDA-nCas9-UGI

**Figure 6.**
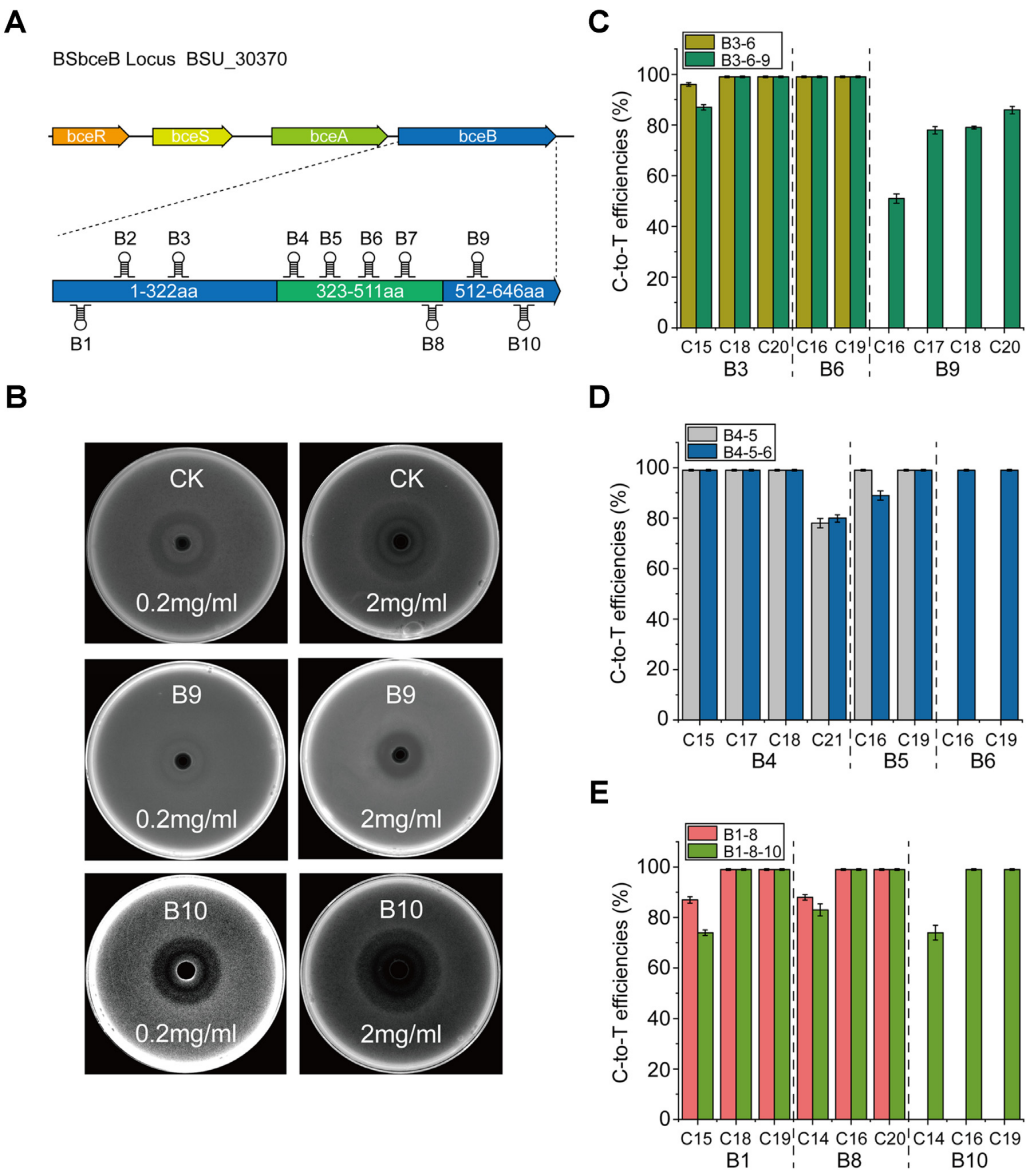
Evolution of BceB by CRISPR-CDA-nCas9-UGI for altering the resistance to bacitracin in *B. subtilis*. (**A**) The position of *bceB* in *bce* gene cluster was shown. Ten sgRNAs were designed to randomly target different positions of the *bceB* gene. (**B**) Antimicrobial assays against wild type (CK) and mutant *B. subtilis* 168 (B9 and B10). Two concentrations (0.2 mg/ml and 2 mg/ml) were used to verify the antimicrobial activity of each strain. (C-E) Eight sgRNAs (B1, B3, B4, B5, B6, B8, B9 and B10) were selected from the above sgRNA library (B1–B10) and randomly combined. Conversion efficiency for multi-sites mutations on *bceB* was calculated. Bars represent the average editing efficiency and error bars represent the S.D. of three independent biological replicates.

To further demonstrate the significant advantages of the CRISPR-CDA-nCas9-UGI, a membrane protein, BceB (coding by *bceB*), was selected as the target protein to evolve. It is a primary bacitracin resistance determinant and also plays an important role in protecting cell wall biosynthetic targets from inhibition by antimicrobial peptides (47,48). A sgRNA library comprising of 10 sgRNAs (B1-B10) was randomly designed and constructed to target the different loci of *bceB* (Figure 6A). Each strain harbouring a specific sgRNA was incubated under several conditions to obtain as many mutants as possible for constructing a mutant library. One clone of each strain was randomly isolated from the mutant library, and bacitracin-resistance analysis was carried out (Figure 6B). Compared with the wild-type *B. subtilis* 168, mutant B9 exhibited higher resistance to bacitracin, while mutant B10 exhibited higher sensitivity to bacitracin (Figure 6B). These results show that the mutants with different sensitivity to bacitracin were successfully obtained via *in situ* mutation. Sequence analysis of the mutants B9 and B10 indicated that the bacitracin-resistance sensitivity of bceB was related with amino acid residues of G552, T624, A625 and L626 (Supplementary Table S5).

The successfully evolution of Sec*-*translocase complex in *B. subtilis* demonstrated that CRISPR-CDA-nCas9-UGI could be used for multi-genes editing simultaneously, to detect whether multi-sites mutation on one gene could occur using the BE, a series sgRNA (three double sgRNAs and three triple sgRNAs) targeting *bceB* gene were designed, and integrated on the *amyE* locus of the *B. subtilis* genome, harbouring CRISPR-CDA-nCas9-UGI. The sequencing results showed that almost of the conversion efficiency of each strain was more than 80%, most of them approached 100%, no matter double sgRNAs and triple sgRNAs were used (Figure 6C–E). These results demonstrate that CRISPR-CDA-nCas9-UGI could also edit multiple sites on one gene simultaneously.

### Detection of off-target editing activity of CRISPR-CDA-nCas9-UGI system

Off-target editing activity is one of the key factors for gene editing (49). To characterize whether CRISPR-CDA-nCas9-UGI system has potential off-target editing activity, five sgRNAs were randomly selected for off-target editing activity detection using Sec*-*translocase complex and BceB as target proteins. The sgRNAs of B3, B4 and B10 were used for *bceB* gene editing, while ET, and GT were used for Sec*-*translocase complex genes editing (Supplementary Figure S5). Each selected sgRNA has some potential off-target sites, which were predicted by Cas-OFFinder (Supplementary Table S6 and Supplementary Figure S5). The genome of base-edited cell was extracted and sequenced by NGS after incubation. The NGS sequencing showed that each sgRNA had high on-target efficiency, but no editing activity was detected at the predicted off-target sites (Supplementary Figure S5). These results certificated that CRISPR-CDA-nCas9-UGI system has almost undetectable off-target editing activity and high on-target editing activity.

## DISCUSSION

Here, we developed a BE CRISPR-CDA-nCas9-UGI exhibiting a high conversion efficiency with an expandable editing window. Compared with other reported BEs, such as CRISPR/Cas9-AID in *Corynebacterium glutamicum* (50), CRISPR-dCas9-AID in *B. subtilis* (32), and dCas-CDA-UL in *E. coli* (31), CRISPR-CDA-nCas9-UGI exhibited high conversion efficiency with a wide editable window. For example, the conversion efficiency of C16 and C19 of the CRISPR/dCas9-AID, another BE in *B. subtilis*, was only 2% and 13%, respectively, and C15 could not be edited at all (32). However, the conversion efficiency of C15, C16, C18 and C19 for the CRISPR-CDA-nCas9-UGI reached 97%, 100%, 100% and 100%, respectively (Figure 3F).

Three reasons may explain the differences in editing efficiency and editable window size among these BEs. First, compared to the dCas9 with no nuclease activity, nCas9 introduced a nick into the unedited single DNA strand, the nicked DNA motivated the DNA repair systems, and DNA tends to use the edited single DNA strand as the template for repair, which facilitates the editing efficiency at the target sites (16–18). Additionally, previous reports showed that the conversion efficiency was sharply reduced when CDA was fused at the C-terminus of nCas9 (17,19), indicating that the position of CDA at the fusion protein influences the editing efficiency. Third, the CDA-nCas9-UGI genes were integrated into the genome of *B. subtilis*, not in a plasmid as done for other BEs, which might maintain the CDA-nCas9-UGI gene expression, increasing the editing functions. Compared with the conversion efficiency of BS1-sigE (harbouring CDA-dCas9 in the genome), the conversion efficiency of a BE using a plasmid harbouring CDA-dCas9 was much lower (data not shown). Additionally, the intergration of the CDA-nCas9-UGI and sgRNA into the genome makes it possible that the base editor may be readily applicable to a wide range of bacteria.

Genome editors are widely applied in genetic engineering, including gene insertions, gene deletions, and point mutations. However, the reported genome editors could not achieve simultaneous multisite mutations with a high efficiency in a wide editable window. CRISPR-CDA-nCas9-UGI, which exhibited a high conversion efficiency with an expanding editable window, could achieve simultaneous multisite mutations with a high efficiency in a wide editable window, which is highly suitable for application in protein evolution. The mutant sites were ensured to be in appointed positions by the designed sgRNAs for targeting genes, avoiding mutants occurring in other genome genes, which are often found in mutants caused by chemical and physical mutagenesis strategies. Therefore, this is a semi-rational strategy for protein evolution, which could significantly increase the evolutionary efficiency. Additionally, the mutants caused by CRISPR-CDA-nCas9-UGI were based on *in situ* mutations *in vivo*, and the state of the environment of the mutants was suitable for function detection, especially for protein complexes, because the function of each protein in the protein complex could not be detected alone without the functional system. Finally, the features exhibited by CRISPR-CDA-nCas9-UGI were suitable for the construction of a mutant library with efficient diversity *in vivo*. The high conversion efficiency enabled high mutational efficiency of the amino acids in target positions; the expanded editable window (8 nt, Figure 3A and B) allowed simultaneous mutations occurring at several sites (the possible four amino acids). Furthermore, the regulatory conversion efficiency by iteration and inducer concentration (Figures 2 and 4), and the different conversion efficiency in the middle and two flanks of the editable window (Figure 2), enabled the construction of a diverse mutant library. These features kept the diversity of the mutants in the mutant library as high as possible, which is beneficial for obtaining target mutants.

The BE of CRISPR-CDA-nCas9-UGI achieved Sec-translocase evolution of *B. subtili*s. This is a successful proof for protein evolution using BE. The Sec translocase is a protein complex composed of SecY, SecE and SecG heterotrimers on the outer membrane. It is difficult to use directed evolution of Sec translocase through error-prone PCR *in vitro* because of the hard expression (large size) and purification (membrane proteins). Additionally, SecY, SecE and SecG in the Sec-translocase complex express their functions in a functional system, which requires the combined actions of each of these and other proteins outside the complex. Even if the mutagenesis *in vitro* is successful, gene substitution *in vivo* has to be conducted subsequently, during which the efficiency of the large-sized gene crossover is low. A lot of protein complex exist in living cell, such as ATP synthase complex, succinate dehydrogenase complex, pyruvate dehydrogenase complex, etc., all of them are important for cellular metabolism. Evolution of the protein complex would helpful for powerful chassis cells construction, however, their evolutions hardly achieve *in vitro*. The BE described here makes it possible to achieve evolutions for these complexes via *in situ* mutation *in vivo*. In addition, a bacitracin-resistance related membrane protein, BceB, was also evolved via *in situ* mutation *in vivo* using BE of CRISPR-CDA-nCas9-UGI. The protein of BceB is a primary bacitracin resistance determinant and plays an important role in protecting cell wall biosynthetic targets from inhibition by antimicrobial peptides (47,48), the bacitracin-resistant mutant is very helpful for constructing a bacitracin-producing cell factory.

It has been reported that a base editing system, CRISPR-X, was developed for gene diversification in mammalian cells (51). The CRISPR-X, it is a very powerful tool for gene diversification as it can tile the entire gene via a sgRNA pool. However, sgRNA pool is hardly introduced in bacterial cells due to the low transformation efficiency and genome repair system in bacterial cells. In fact, diverse combinatorial sgRNAs are more suitable for diversity of the mutants in mutant library, which would be useful for protein evolution, such as the evolutions of the Sec translocase and BceB described in this study. Recently, a tool called TRIDENT has been developed for gene diversification through the fusion of deaminase and T7 RNA polymerase (T7 RNAP) (52). As this tool relies on T7 RNAP-deaminase fusion and the corresponding T7 promoter, a T7 promoter needs to be designed and inserted into the upstream of the target gene, which limits the convenient application for gene editing in the genome. On the contrary, the BE of CRISPR-CDA-nCas9-UGI constructed in this study could edit any genes in the genome with guide of the corresponding sgRNAs. Another base editing system, EvolvR, has been reported to be able to generate mutations using mutated *E. coli* DNA polymerase I (53), however, it is unclear whether this enzyme would have performance in diverse bacteria, especially in Gram-positive strains.

This BE, capable of constructing a protein mutant library efficiently through *in situ* mutation *in vivo*, will contribute to protein evolution, especially the evolution of membrane, toxic and labile proteins, and protein complexes. Additionally, the construction of BE is depended on genome integration, does not rely on any additional or host-dependent factors, indicating that such BEs may be readily constructed and applicable to a wide range of bacteria. The protein evolution information resulted from such BEs would provide guidelines for powerful host cells and chassis cells obtaining through protein engineering. Moreover, as alanine scanning used for functional site detection, the features of targeted and efficient mutation exhibited by CRISPR-CDA-nCas9-UGI could be applied to scan functional genes and functional positions in a functional gene of a genome *in vivo*.

## DATA AVAILABILITY

Raw data of NGS for off-target have been deposited to the NCBI Short Read Archive, with Accession No. BioProject: PRJNA740103 (http://www.ncbi.nlm.nih.gov/bioproject/740103).

## Supplementary Material

gkab673_Supplemental_File

## References

[1] Ho H.I., Fang J.R., Cheung J., Wang H.H. Programmable CRISPR-Cas transcriptional activation in bacteria. Mol. Syst. Biol. 2020; 16:e9427. 32657546 10.15252/msb.20199427 PMC7356669

[2] Miller S.M., Wang T., Randolph P.B., Arbab M., Shen M.W., Huang T.P., Matuszek Z., Newby G.A., Rees H.A., Liu D.R. Continuous evolution of SpCas9 variants compatible with non-G PAMs. Nat. Biotechnol. 2020; 38:471–481. 32042170 10.1038/s41587-020-0412-8 PMC7145744

[3] Voordeckers K., Colding C., Grasso L., Pardo B., Hoes L., Kominek J., Gielens K., Dekoster K., Gordon J., Van der Zande E. et al. Ethanol exposure increases mutation rate through error-prone polymerases. Nat. Commun. 2020; 11:3664. 32694532 10.1038/s41467-020-17447-3 PMC7374746

[4] Krogan N.J., Cagney G., Yu H., Zhong G., Guo X., Ignatchenko A., Li J., Pu S., Datta N., Tikuisis A.P. et al. Global landscape of protein complexes in the yeast *Saccharomyces cerevisiae*. Nature. 2006; 440:637–643. 16554755 10.1038/nature04670

[5] Bao Z., Xiao H., Liang J., Zhang L., Xiong X., Sun N., Si T., Zhao H. Homology-integrated CRISPR-Cas (HI-CRISPR) system for one-step multigene disruption in *Saccharomyces cerevisiae*. ACS Synth. Biol. 2015; 4:585–594. 25207793 10.1021/sb500255k

[6] Wu Y., Chen T., Liu Y., Lv X., Li J., Du G., Amaro R.L., Liu L. CRISPRi allows optimal temporal control of N-acetylglucosamine bioproduction by a dynamic coordination of glucose and xylose metabolism in *Bacillus subtilis*. Metab. Eng. 2018; 49:232–241. 30176395 10.1016/j.ymben.2018.08.012

[7] Jiang Y., Chen B., Duan C., Sun B., Yang J., Yang S. Multigene editing in the *Escherichia coli* genome via the CRISPR-Cas9 system. Appl. Environ. Microbiol. 2015; 81:2506–2514. 25636838 10.1128/AEM.04023-14 PMC4357945

[8] Gao W., Long L., Tian X., Xu F., Liu J., Singh P.K., Botella J.R., Song C. Genome editing in cotton with the CRISPR/Cas9 system. Front Plant Sci. 2017; 8:1364. 28824692 10.3389/fpls.2017.01364 PMC5541054

[9] Hwang W.Y., Fu Y., Reyon D., Maeder M.L., Tsai S.Q., Sander J.D., Peterson R.T., Yeh J.R., Joung J.K. Efficient genome editing in zebrafish using a CRISPR-Cas system. Nat. Biotechnol. 2013; 31:227–229. 23360964 10.1038/nbt.2501 PMC3686313

[10] Jinek M., Chylinski K., Fonfara I., Hauer M., Doudna J.A., Charpentier E. A programmable dual-RNA–guided DNA endonuclease in adaptive bacterial immunity. Science. 2012; 337:820. 10.1126/science.1225829 PMC6286148 22745249

[11] Zetsche B., Gootenberg J.S., Abudayyeh O.O., Slaymaker I.M., Makarova K.S., Essletzbichler P., Volz S.E., Joung J., van der Oost J., Regev A. et al. Cpf1 is a single RNA-guided endonuclease of a class 2 CRISPR-Cas system. Cell. 2015; 163:759–771. 26422227 10.1016/j.cell.2015.09.038 PMC4638220

[12] Chatterjee P., Jakimo N., Lee J., Amrani N., Rodriguez T., Koseki S.R.T., Tysinger E., Qing R., Hao S., Sontheimer E.J. et al. An engineered ScCas9 with broad PAM range and high specificity and activity. Nat. Biotechnol. 2020; 38:1154–1158. 32393822 10.1038/s41587-020-0517-0

[13] Selle K., Barrangou R. Harnessing CRISPR-Cas systems for bacterial genome editing. Trends Microbiol. 2015; 23:225–232. 25698413 10.1016/j.tim.2015.01.008

[14] Westbrook A.W., Moo-Young M., Chou C.P. Development of a CRISPR-Cas9 tool kit for comprehensive engineering of *Bacillus subtilis*. Appl. Environ. Microbiol. 2016; 82:4876–4895. 27260361 10.1128/AEM.01159-16 PMC4968543

[15] Wang Y., Zhang Z.T., Seo S.O., Lynn P., Lu T., Jin Y.S., Blaschek H.P. Bacterial genome editing with CRISPR-Cas9: deletion, integration, single nucleotide modification, and desirable “clean” mutant selection in *Clostridium beijerinckii* as an example. ACS Synth. Biol. 2016; 5:721–732. 27115041 10.1021/acssynbio.6b00060

[16] Nishida K., Arazoe T., Yachie N., Banno S., Kakimoto M., Tabata M., Mochizuki M., Miyabe A., Araki M., Hara H.K. et al. Targeted nucleotide editing using hybrid prokaryotic and vertebrate adaptive immune systems. Science. 2016; 353:aaf8729. 27492474 10.1126/science.aaf8729

[17] Komor A.C., Kim Y.B., Packer M.S., Zuris J.A., Liu D.R. Programmable editing of a target base in genomic DNA without double-stranded DNA cleavage. Nature. 2016; 533:420–424. 27096365 10.1038/nature17946 PMC4873371

[18] Gaudelli N.M., Komor A.C., Rees H.A., Packer M.S., Badran A.H., Bryson D.I., Liu D.R. Programmable base editing of A·T to G·C in genomic DNA without DNA cleavage. Nature. 2017; 551:464–471. 29160308 10.1038/nature24644 PMC5726555

[19] Tan J., Zhang F., Karcher D., Bock R. Engineering of high-precision base editors for site-specific single nucleotide replacement. Nat. Commun. 2019; 10:439. 30683865 10.1038/s41467-018-08034-8 PMC6347625

[20] Xia P.F., Casini I., Schulz S., Klask C.M., Angenent L.T., Molitor B. Reprogramming acetogenic bacteria with CRISPR-targeted base editing via deamination. ACS Synth. Biol. 2020; 9:2162–2171. 32610012 10.1021/acssynbio.0c00226

[21] Wang Y., Liu Y., Li J., Yang Y., Ni X., Cheng H., Huang T., Guo Y., Ma H., Zheng P. et al. Expanding targeting scope, editing window, and base transition capability of base editing in *Corynebacterium glutamicum*. Biotechnol. Bioeng. 2019; 116:3016–3029. 31317533 10.1002/bit.27121

[22] Cheng L., Min D., He R.L., Cheng Z.H., Liu D.F., Yu H.Q. Developing a base-editing system to expand the carbon source utilization spectra of *Shewanella oneidensis* MR-1 for enhanced pollutant degradation. Biotechnol. Bioeng. 2020; 117:2389–2400. 32356906 10.1002/bit.27368

[23] Wang Y., Cheng H., Liu Y., Liu Y., Wen X., Zhang K., Ni X., Gao N., Fan L., Zhang Z. et al. *In-situ* generation of large numbers of genetic combinations for metabolic reprogramming via CRISPR-guided base editing. Nat. Commun. 2021; 12:678. 33514753 10.1038/s41467-021-21003-y PMC7846839

[24] Tong Y., Whitford C.M., Robertsen H.L., Blin K., Jorgensen T.S., Klitgaard A.K., Gren T., Jiang X., Weber T., Lee S.Y. Highly efficient DSB-free base editing for streptomycetes with CRISPR-BEST. Proc. Natl. Acad. Sci. U.S.A. 2019; 116:20366–20375. 31548381 10.1073/pnas.1913493116 PMC6789908

[25] Kuscu C., Parlak M., Tufan T., Yang J., Szlachta K., Wei X., Mammadov R., Adli M. CRISPR-STOP: gene silencing through base-editing-induced nonsense mutations. Nat. Methods. 2017; 14:710–712. 28581493 10.1038/nmeth.4327

[26] Liu Z., Chen M., Chen S., Deng J., Song Y., Lai L., Li Z. Highly efficient RNA-guided base editing in rabbit. Nat. Commun. 2018; 9:2717. 30006570 10.1038/s41467-018-05232-2 PMC6045575

[27] Ma Y., Zhang J., Yin W., Zhang Z., Song Y., Chang X. Targeted AID-mediated mutagenesis (TAM) enables efficient genomic diversification in mammalian cells. Nat. Methods. 2016; 13:1029–1035. 27723754 10.1038/nmeth.4027

[28] Li C., Zong Y., Wang Y., Jin S., Zhang D., Song Q., Zhang R., Gao C. Expanded base editing in rice and wheat using a Cas9-adenosine deaminase fusion. Genome. Biol. 2018; 19:59. 29807545 10.1186/s13059-018-1443-z PMC5972399

[29] Li C., Zhang R., Meng X., Chen S., Zong Y., Lu C., Qiu J.L., Chen Y.H., Li J., Gao C. Targeted, random mutagenesis of plant genes with dual cytosine and adenine base editors. Nat. Biotechnol. 2020; 38:875–882. 31932727 10.1038/s41587-019-0393-7

[30] Zhang Y., Massel K., Godwin I.D., Gao C. Applications and potential of genome editing in crop improvement. Genome Biol. 2018; 19:210. 30501614 10.1186/s13059-018-1586-y PMC6267055

[31] Banno S., Nishida K., Arazoe T., Mitsunobu H., Kondo A. Deaminase-mediated multiplex genome editing in *Escherichia coli*. Nat. Microbiol. 2018; 3:423–429. 29403014 10.1038/s41564-017-0102-6

[32] Yu S., Price M.A., Wang Y., Liu Y., Guo Y., Ni X., Rosser S.J., Bi C., Wang M. CRISPR-dCas9 mediated cytosine deaminase base editing in *Bacillus subtilis*. ACS Synth. Biol. 2020; 9:1781–1789. 32551562 10.1021/acssynbio.0c00151

[33] Anagnostopoulos C., Spizizen J. Requirements for transformation in *Bacillus subtilis*. J. Bacteriol. 1961; 81:741–746. 16561900 10.1128/jb.81.5.741-746.1961 PMC279084

[34] Yan X., Yu H.J., Hong Q., Li S.P. Cre/lox system and PCR-based genome engineering in *Bacillus subtilis*. Appl. Environ. Microbiol. 2008; 74:5556–5562. 18641148 10.1128/AEM.01156-08 PMC2546623

[35] Hao W., Suo F., Lin Q., Chen Q., Zhou L., Liu Z., Cui W., Zhou Z. Design and construction of portable CRISPR-Cpf1-mediated genome editing in *Bacillus subtilis* 168 oriented toward multiple utilities. Front. Bioeng. Biotechnol. 2020; 8:524676. 32984297 10.3389/fbioe.2020.524676 PMC7492563

[36] Labun K., Montague T.G., Krause M., Torres Cleuren Y.N., Tjeldnes H., Valen E. CHOPCHOP v3: expanding the CRISPR web toolbox beyond genome editing. Nucleic Acids Res. 2019; 47:W171–W174. 31106371 10.1093/nar/gkz365 PMC6602426

[37] Hofacker I., Fontana W., Stadler P., Bonhoeffer S., Tacker M., Schuster P. Fast folding and comparison of RNA secondary structures. Monatsh. Chem. 1994; 125:167–188.

[38] Lu Z., Yang S., Yuan X., Shi Y., Ouyang L., Jiang S., Yi L., Zhang G. CRISPR-assisted multi-dimensional regulation for fine-tuning gene expression in *Bacillus subtilis*. Nucleic Acids Res. 2019; 47:e40. 30767015 10.1093/nar/gkz072 PMC6468239

[39] Wang Z., G. S.D., W. M.D Overproduction and characterization of the uracil-DNA glycosylase inhibitor of bacteriophage PBS2. Gene. 1991; 99:31–37. 1902430 10.1016/0378-1119(91)90030-f

[40] Wu Y., Liu Y., Lv X., Li J., Du G., Liu L. CAMERS-B: CRISPR/Cpf1 assisted multiple-genes editing and regulation system for *Bacillus subtilis*. Biotechnol. Bioeng. 2020; 117:1817–1825. 32129468 10.1002/bit.27322

[41] Kluesner M.G., Nedveck D.A., Lahr W.S., Garbe J.R., Abrahante J.E., Webber B.R., Moriarity B.S. EditR: a method to quantify base editing from sanger sequencing. CRISPR J. 2018; 1:239–250. 31021262 10.1089/crispr.2018.0014 PMC6694769

[42] Bae S., Park J., Kim J.S. Cas-OFFinder: a fast and versatile algorithm that searches for potential off-target sites of Cas9 RNA-guided endonucleases. Bioinformatics. 2014; 30:1473–1475. 24463181 10.1093/bioinformatics/btu048 PMC4016707

[43] Song M., Kim H.K., Lee S., Kim Y., Seo S.Y., Park J., Choi J.W., Jang H., Shin J.H., Min S. et al. Sequence-specific prediction of the efficiencies of adenine and cytosine base editors. Nat. Biotechnol. 2020; 38:1037–1043. 32632303 10.1038/s41587-020-0573-5

[44] Zhang S., Feng S., Jiang W., Huang X., Chen J. Construction and optimization of a base editor based on the MS2 system. Anim. Model Exp. Med. 2019; 2:185–190. 10.1002/ame2.12080 PMC6762045 31773094

[45] du Plessis D.J., Nouwen N., Driessen A.J. The Sec translocase. Biochim. Biophys. Acta. 2011; 1808:851–865. 20801097 10.1016/j.bbamem.2010.08.016

[46] Seinen A.B., Driessen A.J.M. Single-molecule studies on the protein translocon. Annu. Rev. Biophys. 2019; 48:185–207. 31084584 10.1146/annurev-biophys-052118-115352

[47] Mascher T., Margulis N.G., Wang T., Ye R.W., Helmann J.D. Cell wall stress responses in *Bacillus subtilis*: the regulatory network of the bacitracin stimulon. Mol. Microbiol. 2003; 50:1591–1604. 14651641 10.1046/j.1365-2958.2003.03786.x

[48] Radeck J., Gebhard S., Orchard P.S., Kirchner M., Bauer S., Mascher T., Fritz G. Anatomy of the bacitracin resistance network in *Bacillus subtilis*. Mol. Microbiol. 2016; 100:607–620. 26815905 10.1111/mmi.13336

[49] Naeem M., Majeed S., Hoque M.Z., Ahmad I. Latest developed strategies to minimize the off-target effects in CRISPR-Cas-mediated genome editing. Cells. 2020; 9:1608. 10.3390/cells9071608 PMC7407193 32630835

[50] Wang Y., Liu Y., Liu J., Guo Y., Fan L., Ni X., Zheng X., Wang M., Zheng P., Sun J. et al. MACBETH: Multiplex automated *Corynebacterium glutamicum* base editing method. Metab. Eng. 2018; 47:200–210. 29580925 10.1016/j.ymben.2018.02.016

[51] Hess G.T., Fresard L., Han K., Lee C.H., Li A., Cimprich K.A., Montgomery S.B., Bassik M.C. Directed evolution using dCas9-targeted somatic hypermutation in mammalian cells. Nat. Methods. 2016; 13:1036–1042. 27798611 10.1038/nmeth.4038 PMC5557288

[52] Cravens A., Jamil O.K., Kong D., Sockolosky J.T., Smolke C.D. Polymerase-guided base editing enables *in vivo* mutagenesis and rapid protein engineering. Nat. Commun. 2021; 12:1579. 33707425 10.1038/s41467-021-21876-z PMC7952560

[53] Halperin S.O., Tou C.J., Wong E.B., Modavi C., Schaffer D.V., Dueber J.E. CRISPR-guided DNA polymerases enable diversification of all nucleotides in a tunable window. Nature. 2018; 560:248–252. 30069054 10.1038/s41586-018-0384-8

