# Supplementary material for: Development of a base editor for protein evolution via in situ mutation in vivo
Source: Nucleic Acids Res. 2021 Aug 14;49(16):9594–605. doi: 10.1093/nar/gkab673 (PMC8450078; doi:10.1093/nar/gkab673)
Supplement: gkab673_Supplemental_File [file gkab673_supplemental_file.pdf]

Supplementary Materials

Development of a base editor for protein evolution via *in situ* mutation *in vivo*

Wenliang Hao[#], Wenjing Cui[#], Zhongyi Cheng, Laichuang Han, Feiya Suo, Zhongmei Liu, Li Zhou, Zhemin Zhou*

[1]The Key Laboratory of Industrial Biotechnology (Ministry of Education), School of Biotechnology, Jiangnan University, 1800 Lihu Avenue, Wuxi 214122, China

Supplementary Information

Supplementary Table S1. The strains used in this study.

Supplementary Table S2. The primers used for gene cloning in this study.

Supplementary Table S3. The plasmids used in this study.

Supplementary Table S4. The gRNA sequences used in this study.

Supplementary Table S5. The mutations of SecYEG and BceB.

Supplementary Table S6. The off-target analysis of CRISPR-CDA-nCas9-UGI

Supplementary Figure S1. Design and construction of multiplex base editing system.

Supplementary Figure S2. Mixed culture sequencing for multiplex base editing in *B. subtilis*.

Supplementary Figure S3. Improvement of editing efficiency by iterating in *B. subtilis*.

Supplementary Figure S4. Sequencing results of the Sec translocase mutants isolated from the mutant library.

Supplementary Figure S5. Off-target editing activities of CRISPR-CDA-nCas9-UGI in *B. subtilis*.

Supplementary Table S1. The strains used in this study.

Strains	Relevant characteristics	References or sources
E. coli JM109	*recA*1, *supE*44 *endA*1 *hsdR*17 (r^{-}k,m^{+}k) *gyrA*96 *relA*1 thi (*lac-proAB*) F'[*traD*36 *proAB*$^{+}$ *lacI*^{q} *lacZ*ΔM15]	Lab stock
B. subtilis 168	*trpC*2	Lab stock
BS1-sigE	*lacA*::P$_{xylA}$-CDA-dCas9, Cm^{R}, harboring pHY-ECBE plasmid.	This study
BS2-sigE	*lacA* :: P$_{xylA}$-CDA-nCas9, Cm^{R}, harboring pHY-ECBE plasmid	This study
BS3-sigE	*lacA* :: P$_{xylA}$-CDA-nCas9, Cm^{R}, harboring pHY-ECBE(Site 1)	This study
BS4-sigE	*lacA* :: P$_{xylA}$-CDA-nCas9, Cm^{R}, harboring pHY-ECBE(Site 2)	This study
BS5-sigE	*lacA* :: P$_{xylA}$-CDA-nCas9, Cm^{R}, harboring pHY-ECBE(Site 3)	This study
BS6-sigE	*lacA* :: P$_{xylA}$-CDA-nCas9, Cm^{R}, harboring pHY-ECBE(Site 4)	This study
BS7	*lacA*::P$_{xylA}$-CDA-nCas9-UGI, Cm^{R}	This study
BS8	*lacA*::P$_{xylA}$-CDA-nCas9-UGI, Cm^{R} , *amyE*::P$_{veg}$-SecY(T1), Spc^{R}	This study
BS9	*lacA*::P$_{xylA}$-CDA-nCas9-UGI, Cm^{R} , *amyE*::P$_{veg}$-	This study

	SecY(T2), Spc^R	
BS10	*lacA*::P_{xylA}-CDA-nCas9-UGI, Cm^R , *amyE*::P_{veg}-SecY(T3), Spc^R	This study
BS11	*lacA*::P_{xylA}-CDA-nCas9-UGI, Cm^R , *amyE*::P_{veg}-SecY(T0)/P_{veg}-SecE(ET), Spc^R	This study
BS12	*lacA*::P_{xylA}-CDA-nCas9-UGI, Cm^R, *amyE*::P_{veg}-SecY(T0)/P_{veg}-SecE(ET)/P_{veg}-SecG(GT), Spc^R	This study
BS13	*lacA*::P_{xylA}-CDA-nCas9-UGI, Cm^R , *amyE*::P_{veg}-SecY(T3)/P_{veg}-SecE(ET), Spc^R	This study
BS14	*lacA*::P_{xylA}-CDA-nCas9-UGI, Cm^R, *amyE*::P_{veg}-SecY(T3)/P_{veg}-SecE(ET)/P_{veg}-SecG(GT), Spc^R	This study
BS15	*lacA*::P_{xylA}-CDA-nCas9-UGI, Cm^R , *amyE*::P_{veg}-bceB(B3)/P_{veg}-bceB(B6), Spc^R	This study
BS16	*lacA*::P_{xylA}-CDA-nCas9-UGI, Cm^R, *amyE*::P_{veg}-bceB(B3)/P_{veg}-bceB(B6)/P_{veg}-bceB(B9), Spc^R	This study
BS17	*lacA*::P_{xylA}-CDA-nCas9-UGI, Cm^R , *amyE*::P_{veg}-bceB(B4)/P_{veg}-bceB(B5), Spc^R	This study
BS18	*lacA*::P_{xylA}-CDA-nCas9-UGI, Cm^R, *amyE*::P_{veg}-bceB(B4)/P_{veg}-bceB(B5)/P_{veg}-bceB(B6), Spc^R	This study
BS19	*lacA*::P_{xylA}-CDA-nCas9-UGI, Cm^R , *amyE*::P_{veg}-bceB(B1)/P_{veg}-bceB(B8), Spc^R	This study
BS20	*lacA*::P_{xylA}-CDA-nCas9-UGI, Cm^R, *amyE*::P_{veg}-	This study

	bceB(B1)/P_{veg}-bceB(B8)/P_{veg}-bceB(B10), Spc^{R}	

Supplementary Table S2. Primers used in this study

Primers	Sequences (5'-3')
pHY-ECBE-F	aaagcctccattatctaaagatggttttagagctagaaatagcaagttaaaataag
pHY-ECBE-R	aaccatctttagataatggaggctttatattttacataatcgcgcgc
pHY-ECBE1-F	gctcccaaacggcgatcagggttttagagctagaaatagcaagttaaaataag
pHY-ECBE1-R	cctgatcgccgtttgggagctttatattttacataatcgcgcgc
pHY-ECBE2-F	tcctcatctttagataatgggttttagagctagaaatagcaagttaaaataag
pHY-ECBE2-R	ccattatctaaagatgaggatttatattttacataatcgcgcgc
pHY-ECBE3-F	aaagcctccattatctaaagatggttttagagctagaaatagcaagttaaaataag
pHY-ECBE3-R	aaccatctttagataatggaggctttatattttacataatcgcgcgc
pHY-ECBE4-F	catcaaaggaaacctctgaagttttagagctagaaatagcaagttaaaataag
pHY-ECBE4-R	ttcagaggtttcctttgatgtttatattttacataatcgcgcgc
pHY-ECBE-21nt-F	gtaaaatataaacgcctccattatctaaagatggttttag
pHY-ECBE-21nt-R	gataatggaggcgtttatattttacataatcgcgcgc
pHY-ECBE-22nt-F	gtaaaatataaaccgcctccattatctaaagatggttttag
pHY-ECBE-22nt-R	gataatggaggcggtttatattttacataatcgcgcgc
pHY-ECBE-23nt-F	gtaaaatataaagccgcctccattatctaaagatggttttag
pHY-ECBE-23nt-R	taatggaggcggctttatattttacataatcgcgcgc
pHY-ECBE-24nt-F	aaaatataaatgccgcctccattatctaaagatggttttag
pHY-ECBE-24nt-R	gataatggaggcggcatttatattttacataatcgcgcgc
pHY-ECBE-25nt-F	gtaaaatataaactgccgcctccattatctaaagatggttttag
pHY-ECBE-25nt-R	atggaggcggcagtttatattttacataatcgcgcgc
pHY-ECBE-26nt-F	gtaaaatataaacctgccgcctccattatctaaagatggttttag
pHY-ECBE-26nt-R	tggaggcggcaggtttatattttacataatcgcgcgc
pHY-ASL-F	gggccgcagccctattgggcaaagcggccctttttcttcagcacaattccaag
pHY-ASL-R	gggccgctttgcccaatagggctgcggcccgcaccgactcggtgcc
pHY-T3-F	ctccttaatacatttggcgggttttagagctagaaatagcaagttaaaataag
pHY-T3-R	ccgccaaatgtattaaggagtttatattttacataatcgcgcgc
pHY-T1-F	atcactcacacgcataaagtgttttagagctagaaatagcaagttaaaataag
pHY-T1-R	actttatgcgtgtgagtgattttatattttacataatcgcgcgc
pHY-T2-F	cctgcaggattcactttcaagttttagagctagaaatagcaagttaaaataag
pHY-T2-R	ttgaaagtgaatcctgcaggtttatattttacataatcgcgcgc
nCDA-F	ggaaatgggatccatgacagatgccgaatacgttc
nCDA-R	tccattctagaagctgcagaaccgacggccgggctcttc
nCDA-b-F	cgtcggttctgcagcttctagaatggataagaaatactcaataggcttag
nCDA-b-R	cggcatctgtcatggatcccatttcccccttttgattttta
pAX-UGI-F	cagctaggaggtgacggaccgaagaagaagcgc
pAX-UGI-R	gatccttactcgagttaaagcattttgatcttattctcgcc

pAX-UGI-b-F	caaaatgctttaactcgagtaaggatctccagg
pAX-UGI-b-R	cttcttcggtccgtcacctcctagctgactcaaa
mAmpR-BsaI-F	atgataccgcgggacccacgctcaccggct
mAmpR-BsaI-R	gtgagcgtgggtcccgcggtatcattgcagcac
secY-F	gttttaccgagtggtctaagcagttttagagctagaaatagcaagttaaaataag
secY-R	aactgcttagaccactcggtaaaacatttattgtacaacacgagcc
SecYT3-F	ctccttaatacatttggcgggttttagagctagaaatagcaagttaaaataag
SecYT3-R	ccgccaaatgtattaaggagacatttattgtacaacacgagcc
secE-F	gtaactgttgaaattaccgtaagttttagagctagaaatagcaagttaaaataag
secE-R	aacttacggtaatttcaacagttacatttattgtacaacacgagcc
secG-F	gtctgccagcactaccgtaatggttttagagctagaaatagcaagttaaaataag
secG-R	aaccattacggtagtgctggcagacatttattgtacaacacgagcc
secY-Go-dual-F	ggtctcatccattattaacgttgatataatttaaattttatttgacaaaaatgg
secY-Go-dual-R	ggtctcgcaccaaaaaagcaccgactcgg
secE-Go-dual-F	ggtctcaggtgttattaacgttgatataatttaaattttatttgacaaaaatgg
secE-Go-dual-R	ggtctcgaagcaaaaaagcaccgactcgg
secE-Go-tri-R	ggtctcgatggaaaaaagcaccgactcgg
secG-Go-tri-F	ggtctcaccatttattaacgttgatataatttaaattttatttgacaaaaatgg
secYE-Go-b-F	ggtctcagcttaagcttgggcttaattaattaagactc
secYE-Go-b-R	ggtctcttggaggatcccgtcgacgc
pHY-B1-F	tccagcaattaacgaagtgagttttagagctagaaatagcaagttaaaataag
pHY-B1-R	tcacttcgttaattgctggatttatattttacataatcgcgcgc
pHY-B2-F	cttcactttgtcttcagtgggttttagagctagaaatagcaagttaaaataag
pHY-B2-R	ccactgaagacaaagtgaagtttatattttacataatcgcgcgc
pHY-B3-F	cgcaacaaacagttcattaagttttagagctagaaatagcaagttaaaataag
pHY-B3-R	ttaatgaactgtttgttgcgtttatattttacataatcgcgcgc
pHY-B4-F	agccgctacattttgttcaggttttagagctagaaatagcaagttaaaataag
pHY-B4-R	ctgaacaaaatgtagcggcttttatattttacataatcgcgcgc
pHY-B5-F	tcaacatttgcttgcaggacgttttagagctagaaatagcaagttaaaataag
pHY-B5-R	gtcctgcaagcaaatgttgatttatattttacataatcgcgcgc
pHY-B6-F	acagcaagctgcatattgccgttttagagctagaaatagcaagttaaaataag
pHY-B6-R	ggcaatatgcagcttgctgttttatattttacataatcgcgcgc
pHY-B7-F	gcccccgcttgtaaacgtgtgttttagagctagaaatagcaagttaaaataag
pHY-B7-R	acacgtttacaagcgggggctttatattttacataatcgcgcgc
pHY-B8-F	ctggcgcaatctacatttatgttttagagctagaaatagcaagttaaaataag
pHY-B8-R	ataaatgtagattgcgccagtttatattttacataatcgcgcgc
pHY-B9-F	cacccatttgtttaaaataagttttagagctagaaatagcaagttaaaataag
pHY-B9-R	ttattttaaacaaatgggtgtttatattttacataatcgcgcgc
pHY-B10-F	actgcgctctactccattttgttttagagctagaaatagcaagttaaaataag
pHY-B10-R	aaaatggagtagagcgcagttttatattttacataatcgcgcgc
pAD-B1-F	tccagcaattaacgaagtgagttttagagctagaaatagcaagttaaaataag
pAD-B1-R	tcacttcgttaattgctggaacatttattgtacaacacgagcc
pAD-B3-F	cgcaacaaacagttcattaagttttagagctagaaatagcaagttaaaataag

pAD-B3-R	ttaatgaactgtttgttgcgacatttattgtacaacacgagcc
pAD-B4-F	agccgctacattttgttcaggttttagagctagaaatagcaagttaaaataag
pAD-B4-R	ctgaacaaaatgtagcggctacatttattgtacaacacgagcc
pAD-B5-F	tcaacatttgcttgcaggacgttttagagctagaaatagcaagttaaaataag
pAD-B5-R	gtcctgcaagcaaatgttgaacatttattgtacaacacgagcc
pAD-B6-F	acagcaagctgcatattgccgttttagagctagaaatagcaagttaaaataag
pAD-B6-R	ggcaatatgcagcttgctgtacatttattgtacaacacgagcc
pAD-B8-F	ctggcgcaatctacatttatgttttagagctagaaatagcaagttaaaataag
pAD-B8-R	ataaatgtagattgcgccagacatttattgtacaacacgagcc
pAD-B9-F	cacccatttgtttaaaataagttttagagctagaaatagcaagttaaaataag
pAD-B9-R	ttattttaaacaaatgggtgacatttattgtacaacacgagcc
pAD-B10-F	actgcgctctactccattttgttttagagctagaaatagcaagttaaaataag
pAD-B10-R	aaaatggagtagagcgcagtacatttattgtacaacacgagcc

Supplementary Table S3. The plasmids used in this study

Plasmids	Relevant characteristics	References or sources
pHYT-P43-G10	*E.coli-B.subtilis* shuttle vector，P43 promoter，gRNA targeting GFP，Amp^R，Tet^R	Lu et al., 2019 (1)
pDGT-GFP	*B. subtilis* integration vector, P43-GFP cassette，$spec^R$	Lu et al., 2019 (1)
pDGT-GFP-Amp_m	Derived from pDGT-GFP, P43-GFP cassette，synonymous mutation in AmpR (TC<u>T</u>240TC<u>C</u>), $spec^R$	This study
pAX01-Cpf1	*B. subtilis* integration vector，xylR-P_{xyl}-Cpf1 cassette，$spec^R$，Cm^R	Lab stock
pAX-nCas9	*B. subtilis* integration vector，xylR-P_{xyl}-nCas9 cassette，$spec^R$，Cm^R	Lab stock
pAX-dCas9	*B. subtilis* integration vector，xylR-P_{xyl}-dCas9 cassette，$spec^R$，Cm^R	Lab stock
pHY-ECBE	Derived from pHYT-P43-G10, gRNA targeting *sigE*	This study
pAD-T0	pAD123 derivative，P_{veg} promoter, containing gRNA expression cassette targeting *secY*	This study
pAD-T3	pAD123 derivative，P_{veg} promoter,	This study

	containing gRNA expression cassette targeting *secY*(T3)	
pAD-secE	pAD123 derivative，P_{veg} promoter, containing gRNA expression cassette targeting *secE*	This study
pAD-secG	pAD123 derivative，P_{veg} promoter, containing gRNA expression cassette targeting *secG*	This study
pDGT-T1	pDGT-GFP derivative, P_{veg} promoter, containing gRNA expression cassette targeting *secY*(T1)	This study
pDGT-T2	pDGT-GFP derivative, P_{veg} promoter, containing gRNA expression cassette targeting *secY*(T2)	This study
pDGT-T3	pDGT-GFP derivative, P_{veg} promoter, containing gRNA expression cassette targeting *secY*(T3)	This study
pDGT-T0ET	pDGT-GFP derivative, P_{veg} promoter, containing gRNA expression cassette targeting *secY* and *secE*	This study
pDGT-T0ETGT	pDGT-GFP derivative, P_{veg} promoter, containing gRNA expression cassette targeting *secY*, *secE* and *secG*	This study
pDGT-T3ET	pDGT-GFP derivative, P_{veg} promoter, containing gRNA expression cassette targeting *secY*(T3) and *secE*(ET)	This study
pDGT-T3ETGT	pDGT-GFP derivative, P_{veg} promoter, containing gRNA expression cassette targeting *secY*(T3), *secE*(ET) and *secG*(GT)	This study
pAD-B1	pAD123 derivative，P_{veg} promoter, containing gRNA expression cassette targeting *bceB*(B1)	This study
pAD-B3	pAD123 derivative，P_{veg} promoter, containing gRNA expression cassette targeting *bceB*(B3)	This study
pAD-B4	pAD123 derivative，P_{veg} promoter, containing gRNA expression	This study

	cassette targeting *bceB*(B4)	
pAD-B5	pAD123 derivative，P_{veg} promoter, containing gRNA expression cassette targeting *bceB*(B5)	This study
pAD-B6	pAD123 derivative，P_{veg} promoter, containing gRNA expression cassette targeting *bceB*(B6)	This study
pAD-B8	pAD123 derivative，P_{veg} promoter, containing gRNA expression cassette targeting *bceB*(B8)	This study
pAD-B9	pAD123 derivative，P_{veg} promoter, containing gRNA expression cassette targeting *bceB*(B9)	This study
pAD-B10	pAD123 derivative，P_{veg} promoter, containing gRNA expression cassette targeting *bceB*(B10)	This study
pDGT-B36	pDGT-GFP derivative, P_{veg} promoter, containing gRNA expression cassette targeting *bceB*(B3) and *bceB*(B6)	This study
pDGT-B369	pDGT-GFP derivative, P_{veg} promoter, containing gRNA expression cassette targeting *bceB*(B3), *bceB*(B6) and *bceB*(B9)	This study
pDGT-B45	pDGT-GFP derivative, P_{veg} promoter, containing gRNA expression cassette targeting *bceB*(B4) and *bceB*(B5)	This study
pDGT-B456	pDGT-GFP derivative, P_{veg} promoter, containing gRNA expression cassette targeting *bceB*(B4), *bceB*(B5) and *bceB*(B6)	This study
pDGT-B18	pDGT-GFP derivative, P_{veg} promoter, containing gRNA expression cassette targeting *bceB*(B1) and *bceB*(B8)	This study
pDGT-B1810	pDGT-GFP derivative, P_{veg} promoter, containing gRNA expression cassette targeting *bceB*(B1), *bceB*(B8) and *bceB*(B10)	This study
pAX-CDA-dCas9	pAX01-Cpf1 derivative, P_{xylA}-CDA-dCas9 expression cassette, $spec^R$， Cm^R	This study

pAX-CDA-nCas9	pAX-CDA-dCas9 derivative, P_{xylA}-CDA-nCas9 expression cassette, $spec^R$, Cm^R	This study
pAX-CDA-nCas9-UGI	pAX-CDA-nCas9 derivative, P_{xylA}-CDA-nCas9-UGI expression cassette, $spec^R$, Cm^R	This study
pB-P_{srfA}-WapA-GFP	*E.coli-B.subtilis* shuttle vector, containing P_{srfA}-WapA-GFP expression cassette, Amp^R, Kan^R	This study
pHT-ETGT	*E.coli-B.subtilis* shuttle vector, containing xylR-P_{xylA}-CDA-nCas9-UGI expression cassette and gRNA expression cassette targeting *secE*(ET) and *secG*(GT), Amp^R, Cm^R	This study

Supplementary Table S4. sgRNAs used in this study

sgRNA Sequences (5'-3')	PAM	Purpose
(GCCTCCATTATCTAAAGATG)[a]GTTTTAGAGCTAGAAATAGCAAGTTAAAATAAGGCTAGTCCGTTAT(CAACTTGAAAAAGTGGCACCGAGTCGGTGCTTTTTT)[b]	AGG	Targeting *sigE* (20nt)
(GCCTCCATTATCTAAAGATG)[a]GTTTTAGAGCTAGAAATAGCAAGTTAAAATAAGGCTAGTCCGTTAT(CAACTTGAAAAAGTGGCACCGAGTCGGTGC)[b](GGGCCGCAGCCCTATTGGGCAAAGCGGCCC)[c]TTTTTT[b]	AGG	Targeting *sigE* (20nt)
(CGCCTCCATTATCTAAAGATG)[a]GTTTTAGAGCTAGAAATAGCAAGTTAAAATAAGGCTAGTCCGTTAT(CAACTTGAAAAAGTGGCACCGAGTCGGTGCTTTTTT)[b]	AGG	Targeting *sigE* (21nt)
(CCGCCTCCATTATCTAAAGATG)[a]GTTTTAGAGCTAGAAATAGCAAGTTAAAATAAGGCTAGTCCGTTAT(CAACTTGAAAAAGTGGCACCGAGTCGGTGCTTTTTT)[b]	AGG	Targeting *sigE* (22nt)
(GCCGCCTCCATTATCTAAAGATG)[a]GTTTTAGAGCTAGAAATAGCAAGTTAAAATAAGGCTAGTCCGTTAT(CAAC	AGG	Targeting *sigE* (23nt)

TTGAAAAAGTGGCACCGAGTCGGT GCTTTTTT)[b]		
(TGCCGCCTCCATTATCTAAAGATG)[a] GTTTTAGAGCTAGAAATAGCAAGTT AAAATAAGGCTAGTCCGTTAT(CAA CTTGAAAAAGTGGCACCGAGTCGGT GCTTTTTT)[b]	AGG	Targeting *sigE* (24nt)
(CTGCCGCCTCCATTATCTAAAGATG)[a]GTTTTAGAGCTAGAAATAGCAAGT TAAAATAAGGCTAGTCCGTTAT(CA ACTTGAAAAAGTGGCACCGAGTCG GTGCTTTTTT)[b]	AGG	Targeting *sigE* (25nt)
(CCTGCCGCCTCCATTATCTAAAGAT G)[a]GTTTTAGAGCTAGAAATAGCAAG TTAAAATAAGGCTAGTCCGTTAT(C AACTTGAAAAAGTGGCACCGAGTC GGTGCTTTTTT)[b]	AGG	Targeting *sigE* (26nt)
(GCTCCCAAACGGCGATCAGG)[a]GTT TTAGAGCTAGAAATAGCAAGTTAA AATAAGGCTAGTCCGTTAT(CAACTT GAAAAAGTGGCACCGAGTCGGTGC TTTTTT)[b]	CGG	Targeting *sigE* (Site 1)
(TCCTCATCTTTAGATAATGG)[a]GTTT TAGAGCTAGAAATAGCAAGTTAAA ATAAGGCTAGTCCGTTAT(CAACTTG AAAAAGTGGCACCGAGTCGGTGCTT TTTT)[b]	AGG	Targeting *sigE* (Site 2)
(GCCTCCATTATCTAAAGATG)[a]GTTT TAGAGCTAGAAATAGCAAGTTAAA ATAAGGCTAGTCCGTTAT(CAACTTG AAAAAGTGGCACCGAGTCGGTGCTT TTTT)[b]	AGG	Targeting *sigE* (Site 3)
(CATCAAAGGAAACCTCTGAA)[a]GTT TTAGAGCTAGAAATAGCAAGTTAA AATAAGGCTAGTCCGTTAT(CAACTT GAAAAAGTGGCACCGAGTCGGTGC TTTTTT)[b]	CGG	Targeting *sigE* (Site 4)
(TTTACCGAGTGGTCTAAGCA)[a]GTTT TAGAGCTAGAAATAGCAAGTTAAA ATAAGGCTAGTCCGTTAT(CAACTTG AAAAAGTGGCACCGAGTCGGTGCTT TTTT)[b]	AGG	Targeting *secY*(T0)
(ATCACTCACACGCATAAAGT)[a]GTTT TAGAGCTAGAAATAGCAAGTTAAA	TGG	Targeting *secY*(T1)

ATAAGGCTAGTCCGTTAT(CAACTTGAAAAAGTGGCACCGAGTCGGTGCTTTTTT)[b]		
(CCTGCAGGATTCACTTTCAA)[a]GTTTTAGAGCTAGAAATAGCAAGTTAAAATAAGGCTAGTCCGTTAT(CAACTTGAAAAAGTGGCACCGAGTCGGTGCTTTTTT)[b]	TGG	Targeting *secY*(T2)
(CTCCTTAATACATTTGGCGG)[a]GTTTTAGAGCTAGAAATAGCAAGTTAAAATAAGGCTAGTCCGTTAT(CAACTTGAAAAAGTGGCACCGAGTCGGTGCTTTTTT)[b]	CGG	Targeting *secY*(T3)
(AACTGTTGAAATTACCGTAA)[a]GTTTTAGAGCTAGAAATAGCAAGTTAAAATAAGGCTAGTCCGTTAT(CAACTTGAAAAAGTGGCACCGAGTCGGTGCTTTTTT)[b]	TGG	Targeting *secE*
(CTGCCAGCACTACCGTAATG)[a]GTTTTAGAGCTAGAAATAGCAAGTTAAAATAAGGCTAGTCCGTTAT(CAACTTGAAAAAGTGGCACCGAGTCGGTGCTTTTTT)[b]	CGG	Targeting *secG*
(TCCAGCAATTAACGAAGTGA)[a]GTTTTAGAGCTAGAAATAGCAAGTTAAAATAAGGCTAGTCCGTTAT(CAACTTGAAAAAGTGGCACCGAGTCGGTGCTTTTTT)[b]	AGG	Targeting *bceB*(B1)
(CTTCACTTTGTCTTCAGTGG)[a]GTTTTAGAGCTAGAAATAGCAAGTTAAAATAAGGCTAGTCCGTTAT(CAACTTGAAAAAGTGGCACCGAGTCGGTGCTTTTTT)[b]	AGG	Targeting *bceB*(B2)
(CGCAACAAACAGTTCATTAA)[a]GTTTTAGAGCTAGAAATAGCAAGTTAAAATAAGGCTAGTCCGTTAT(CAACTTGAAAAAGTGGCACCGAGTCGGTGCTTTTTT)[b]	TGG	Targeting *bceB*(B3)
(AGCCGCTACATTTTGTTCAG)[a]GTTTTAGAGCTAGAAATAGCAAGTTAAAATAAGGCTAGTCCGTTAT(CAACTTGAAAAAGTGGCACCGAGTCGGTGCTTTTTT)[b]	CGG	Targeting *bceB*(B4)
(TCAACATTTGCTTGCAGGAC)[a]GTTT	AGG	Targeting *bceB*(B5)

TAGAGCTAGAAATAGCAAGTTAAA ATAAGGCTAGTCCGTTAT(CAACTTG AAAAAGTGGCACCGAGTCGGTGCTT TTTT)[b]		
(ACAGCAAGCTGCATATTGCC)[a]GTTT TAGAGCTAGAAATAGCAAGTTAAA ATAAGGCTAGTCCGTTAT(CAACTTG AAAAAGTGGCACCGAGTCGGTGCTT TTTT)[b]	GGG	Targeting *bceB*(B6)
(GCCCCCGCTTGTAAACGTGT)[a]GTTT TAGAGCTAGAAATAGCAAGTTAAA ATAAGGCTAGTCCGTTAT(CAACTTG AAAAAGTGGCACCGAGTCGGTGCTT TTTT)[b]	AGG	Targeting *bceB*(B7)
(CTGGCGCAATCTACATTTAT)[a]GTTT TAGAGCTAGAAATAGCAAGTTAAA ATAAGGCTAGTCCGTTAT(CAACTTG AAAAAGTGGCACCGAGTCGGTGCTT TTTT)[b]	CGG	Targeting *bceB*(B8)
(CACCCATTTGTTTAAAATAA)[a]GTTT TAGAGCTAGAAATAGCAAGTTAAA ATAAGGCTAGTCCGTTAT(CAACTTG AAAAAGTGGCACCGAGTCGGTGCTT TTTT)[b]	AGG	Targeting *bceB*(B9)
(ACTGCGCTCTACTCCATTTT)[a]GTTT TAGAGCTAGAAATAGCAAGTTAAA ATAAGGCTAGTCCGTTAT(CAACTTG AAAAAGTGGCACCGAGTCGGTGCTT TTTT)[b]	TGG	Targeting *bceB*(B10)

Annotations:

[a]: 20-26 nt base target site
[b]: tracr RNA terminator
[c]: artificial stem loop

Supplementary Table S5. The mutations of SecYEG and BceB

Mutants	Position of mutation
SecY(T1)	M9I/S12N/D13N
SecY(T2)	A269T/G270K/V271I
SecY(T3)	L55F/L56F
SecY/SecE(T3/ET)	SecY(L55F/L56F)/SecE(V36I)
SecY/SecE/SecG(T3/ET/GT)	SecY(L55F/L56F)/SecE(V36I)/SecG(A62T/V63I)

SecE/SecG(ET/GT)	SecE(V36I)/SecG(A62T/V63I)
bceB(B1)	P42L/A43V
bceB(B2)	V194I
bceB(B3)	V235I/A236T
bceB(B4)	A326T/A327T/D328N
bceB(B5)	V363I/D364N
bceB(B6)	A386T/V387I
bceB(B7)	S452N/G453K/G454N
bceB(B8)	A479V/Q480*
bceB(B9)	G552S
bceB(B10)	T624I/A625V/L626F

Supplementary Table S6. The off-target analysis of CRISPR-CDA-nCas9-UGI in *B. subtilis* 168

Loci & target sequence (5'-3')	similar DNA sequences[a]	Positions[b]	Mismatches[c]	Positive[d]
bceB(B3) CGCAACAAACAGTTCATTAATGG	gGCttCAAACAGTTCATgAA	3120467	4	N. D.
	CGaAACAAtCgGTgtATTAA	658364	5	N. D.
	CGCAAaAcAaAGcTCcTgAA	360677	6	N. D.
	CGCAgCAAAttcaTCtTTAA	443778	6	N. D.
	gGCAtCAAAgAGTaCAccAA	591691	6	N. D.
	CGCAcCAtgCtGTTgATaAA	793200	6	N. D.
	CGgggCAAtCAtTTCATTtA	1263495	6	N. D.
	aaCAACAAAgAGgTCATTgc	1609401	6	N. D.
	CtgAtCAAACAaTTCAaTgA	1179261	6	N. D.
	tGggACAAAtttTTCATTAA	1525202	6	N. D.
	gaCAACAAACtGTcCATTgt	1525575	6	N. D.
	gGCtAgAAACAGaTCATatA	1901709	6	N. D.

	tGaAgaAAACcGTTtATTAA	2122392	6	N. D.
	CGCcAaAAAgAaTTgATaAA	2945681	6	N. D.
	gaaAAaAAACAGcTCATcAA	3098482	6	N. D.
	CGCcAaAAAttGaTCATgAA	3185661	6	N. D.
	CGCtgCAAgCgGcTtATTAA	3235765	6	N. D.
	CGgAAgcAAaAaTaCATTAA	3626871	6	N. D.
	gGCttCAAACAGcTCtTgAA	3738711	6	N. D.
	CGCAAgAAtCAGgTtAcTgA	3853065	6	N. D.
bceB(B4) AGCCGCTACATTTTGTTCAGCGG	gtCtGCgtCtTTTTGTTCAG	761694	6	N. D.
	AGCaGagAaATTTTGTTtgG	831289	6	N. D.
	AGCCGCTACAccagcTTCAa	509561	6	N. D.
	AGgCGCagCATTTacTTCAa	284161	6	N. D.
	tttgGCgtCATTTTGTTCAG	613248	6	N. D.
	AGCCcCTgacTagTGTTCAG	288622	6	N. D.
	AaCgaCTggATTgTGTTCAG	292295	6	N. D.
	AcCgGCaAtATTTTGccCAG	939589	6	N. D.
	AaCCGgTAttTTTTGTgCAt	1236819	6	N. D.
	AGCttCagCATTTTcTTgAG	1357073	6	N. D.
	AGCCaCTcCtTcTgtTTCAG	1392805	6	N. D.
	AGCttCTgCAaTTTGTatAG	1658479	6	N. D.
	AGtCtCTtCATTTgGTgCcG	1814963	6	N. D.
	gGCtGCTgCATTTTaTcCAa	1894768	6	N. D.

	AGCCGgTACtaTgTtTTtAG	1887497	6	N. D.
	AtCCctTACcTTTgGTTCtG	2098160	6	N. D.
	AaaCGCaAaATTgcGTTCAG	2200181	6	N. D.
	tGCCGCaACAgTTaGTaCAc	2404983	6	N. D.
	AcCgtCTACATcTTtaTCAG	2528892	6	N. D.
	AGCgGCTACAgTgTGgctAG	3242693	6	N. D.
	AGCCGgTACATTcaGTgacG	3286788	6	N. D.
	AGCCGCagCATTTTGaaaAc	3198856	6	N. D.
	AaCaaCaACATTTTcTTCAa	3351009	6	N. D.
	AGCCGtTgtcTTTTtTcCAG	3280087	6	N. D.
	gGgCGCTgCtTTTTGTTCtt	3792635	6	N. D.
	AGCgGCTgCtaTTTGgTCgG	4003257	6	N. D.
	AatCtaTcCATTTTGTTCcG	4056053	6	N. D.
bceB(B10) ACTGCGCTCTACTCCATTTTTGG	ACTcaGCTtTcCgCCATTTT	1384242	5	N. D.
	AaTGCGCTaTtCcgCAaTTT	1319511	6	N. D.
	ttTcCcCTCTAaTCCtTTTT	862082	6	N. D.
	AtTGaGCTCTtCTgCtTTTc	1327945	6	N. D.
	taTGttCTCTcCTCtATTTT	1709986	6	N. D.
	ACTtCGaTtTtCgCgATTTT	1676275	6	N. D.
	ACaGCGgaCgAagCCATTTT	1759907	6	N. D.
	ACTGCtaTacACTCCtTaTT	1894393	6	N. D.
	ctTGCGCcCTACTCgtTTTg	2160530	6	N. D.

	ACgGaGCTtTgCTtCAcTTT	2272339	6	N. D.
	AaTcCttTCTAaTCCtTTTT	3053355	6	N. D.
	gCTGCGgaCaACgCaATTTT	3149300	6	N. D.
	ACTGCcCTgTACcgCATTga	3220052	6	N. D.
	ACTcCGCTCcAgTCtgTaTT	3587863	6	N. D.
	caTGCttTCTcCTCCATTTc	3979194	6	N. D.
	AaTcCGCTCTAaTCCccTTc	4198187	6	N. D.
secE(ET)<br><br>AACTGTTGAAATTACCGTAATGG	AAgTGTTGAAATTgCgagAA	317407	5	N. D.
	cACTGTatAAAaTgCCGTAA	1696780	5	N. D.
	AACaGcTaAAATTACCGaAc	1866534	5	N. D.
	AttTtcTGAAATgACCGTAA	3988157	5	N. D.
	AAaTGTacAccTTtCCGTAA	220164	6	N. D.
	AACTGaTGAAAaTcaCGaAg	606068	6	N. D.
	AgCgGTTGAAAaaACgGTcA	472034	6	N. D.
	AACTGTTGtAcTTgCttTAg	562905	6	N. D.
	AtCaGTTGAAtcTACaaTAA	567300	6	N. D.
	AACTGTTGtcATTcCCaacA	1024286	6	N. D.
	AAaaGcTGAAATTgCCagAA	889421	6	N. D.
	tgCTGgTGAAATatCCGTcA	1067619	6	N. D.
	cACaGTTGAAATTcCCaTct	2012454	6	N. D.
	AACTGTTGtAAgcAgCGTcc	2124900	6	N. D.
	AACaGcTGAAATTgCgGTgc	2559395	6	N. D.

	tcCTaTTtAcATTACtGTAA	2678358	6	N. D.
	AAaTGaTGAAATTgCCcTtg	2570871	6	N. D.
	AAgTGcaGtAATTtCCGTgA	3106624	6	N. D.
	AgCaacTGAAATgAtCGTAA	3355687	6	N. D.
	AtCTaTTGAAgcaACCGTtA	3390424	6	N. D.
	AtCTGaaGAAATggCaGTAA	3545382	6	N. D.
	AcCTcTTGAAATgACaGTtc	4057929	6	N. D.
secG(GT) CTGCCAGCACTACCGTAATGCGG	CTcCCAGCACTAtgtTgATG	3023041	5	N. D.
	tTGCCAGtAtcACCGTAAcc	690206	6	N. D.
	CTGCCAtttCTACgGTgAcG	901205	6	N. D.
	CaGCCAGCACaAgCcTgATc	1820492	6	N. D.
	tTGCCAGCACTtCaaTgAgG	1773765	6	N. D.
	CaaCgAGCtCTACCtTAATc	2468172	6	N. D.
	CTGaCAGaACTACaGTAtca	2827558	6	N. D.
	CaGCtAGCACTgCaGaAAcG	3475794	6	N. D.

Annotations:

[a]: Choromosomal areas most similar to target sequence were predicted by Cas-OFFinder
[b]: Genome positions of *Bacillus subtilis* 168 predicted by Cas-OFFinder
[c]: The number of mismatched bases between target region and off-target region
[d]: The designated off-target Positions were detected by Next-Generation Sequencing (NGS)
N.D.: Not detection

Supplementary Figure

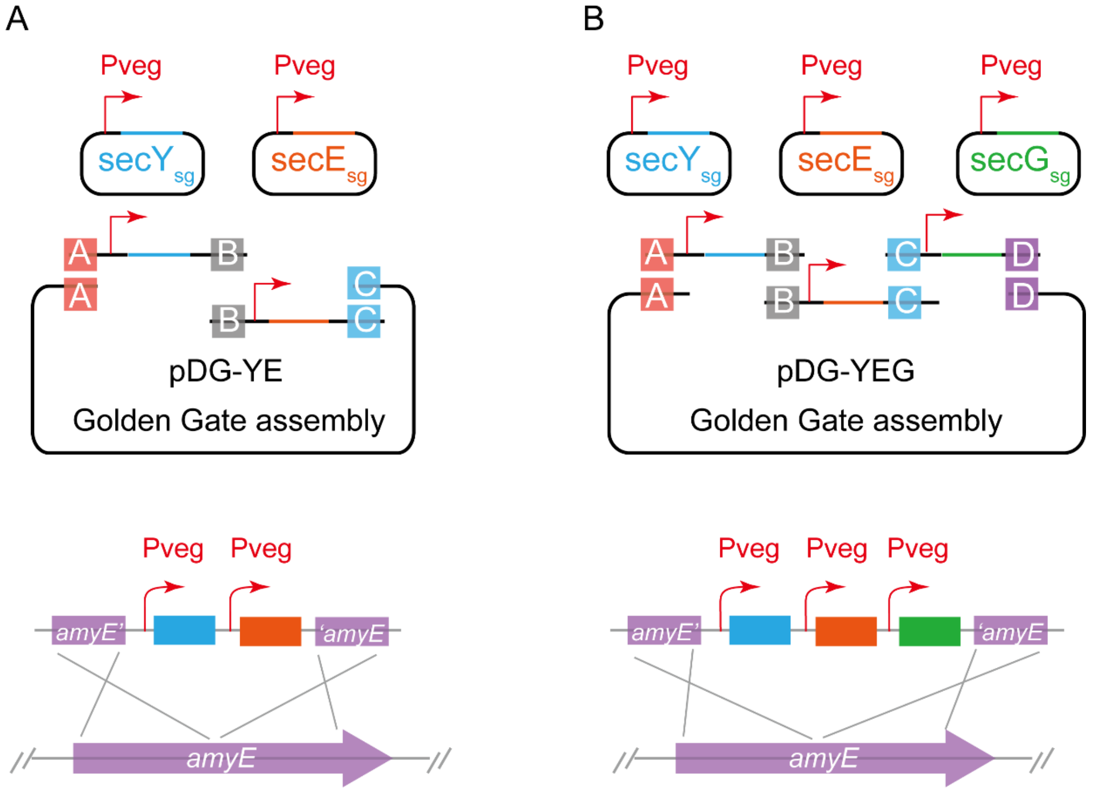

Supplementary Figure S1. Design and construction of multiplex base editing system. (A) For double genes editing, the sgRNA targeting *secY*, and *secE* were simultaneously ligated to the integration vector PDG via Golden Gate assembly. The resulted pDG-YE was linearized and integrated into the *amyE* site of *B. subtilis* genome. (B) For triplex genes editing, the sgRNA targeting *secY*, *secE*, and *secG* were simultaneously ligated to the integration vector PDG via Golden Gate assembly. The resulted pDG-YEG was linearized and integrated into the *amyE* site of *B. subtilis* genome.

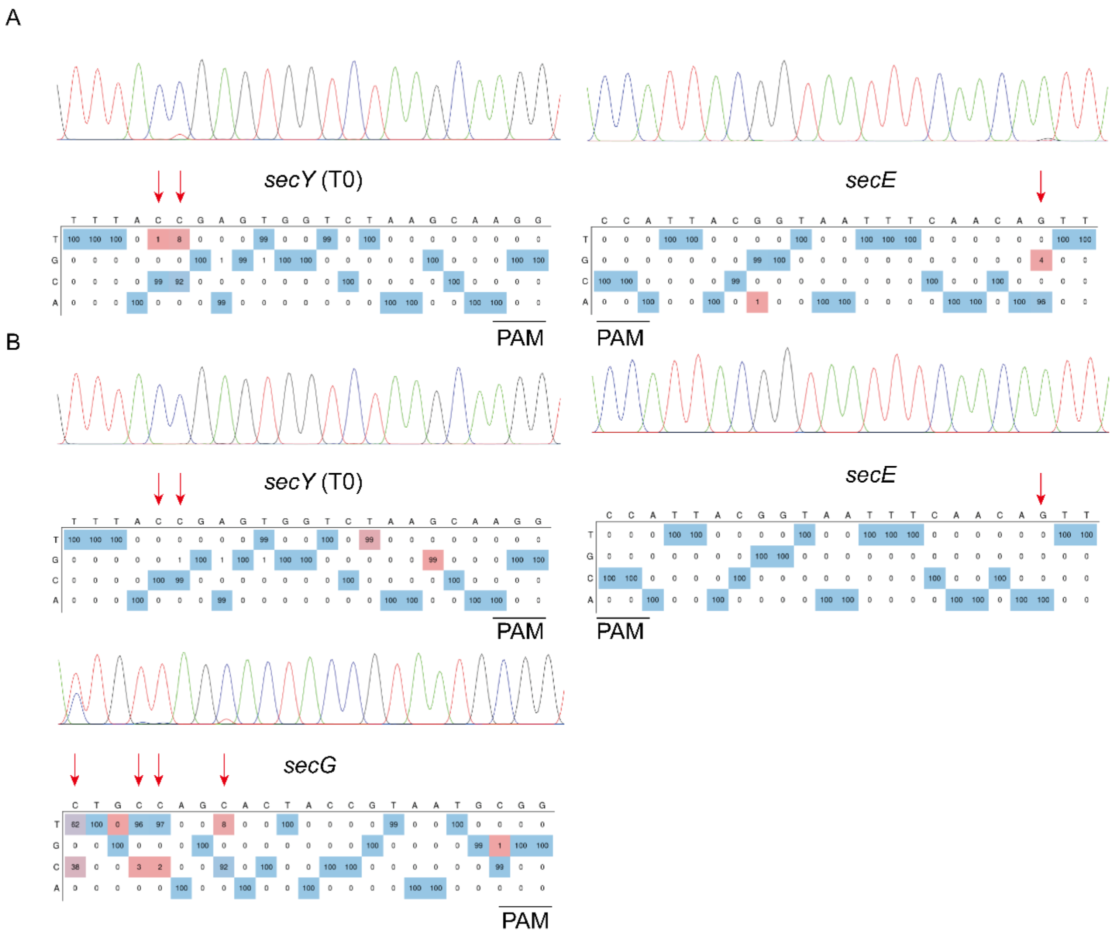

Supplementary Figure S2. Mixed culture sequencing for multiplex base editing in *B. subtilis*. (A) The sequencing results concurrently edited by *secY* and *secE*. (B) The sequencing results concurrently edited by *secY*, *secE* and *secG*.

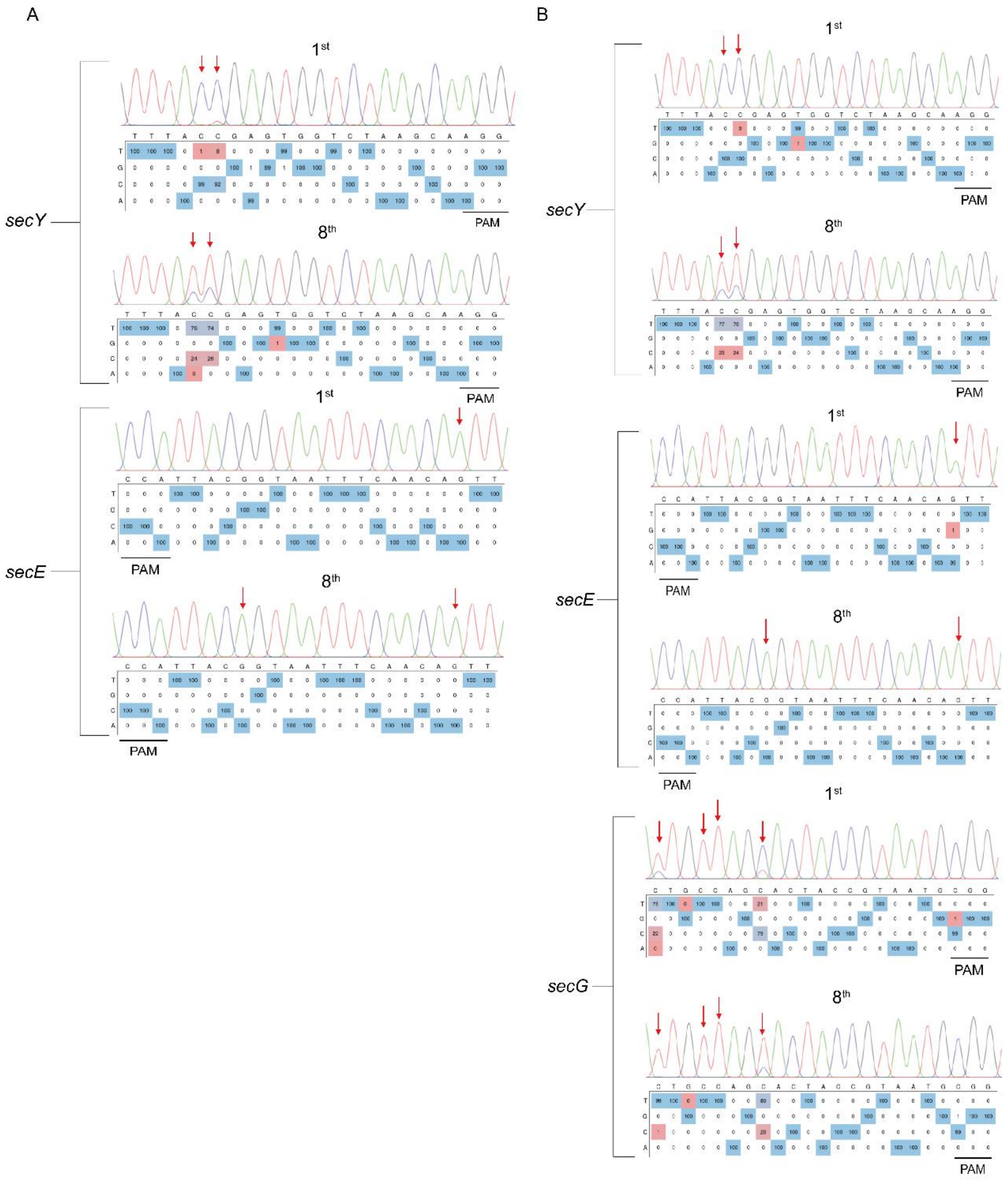

Supplementary Figure S3. Improvement of editing efficiency by iterating in *B. subtilis*. (A) The population sequencing results for editing secY and secE simultaneously. The first and eighth generation sequencing results were displayed from top to bottom. (B) The population sequencing results for editing secY, secE and secG simultaneously. The first and eighth generation sequencing results were displayed from top to bottom. The red arrow indicates the modified bases.

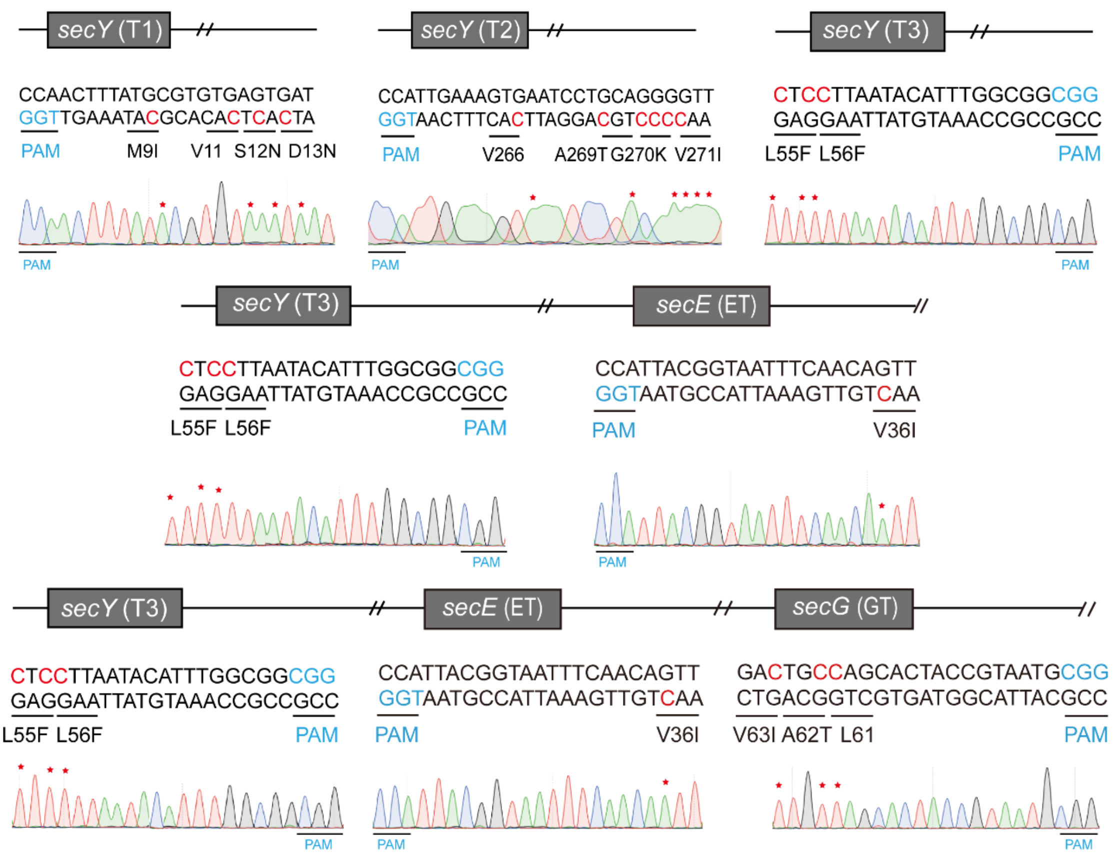

Supplementary Figure S4. Sequencing results of the Sec translocase mutants isolated from the mutant library. After about 10 hours for base editing, each base-edited cell culture was diluted to a suitable concentration and evenly spread on the LB plates. Two colonies were randomly selected from the above LB plates for sequencing. Different mutants of *secY* were shown at the top. The combined mutant of *secY* and *secE* was shown in the middle. The triple combined mutants of *secY*, *secE*, and *secG* were shown at the bottom. The red stars indicate the position of the expected mutation. The substituted amino acids were shown in each mutant.

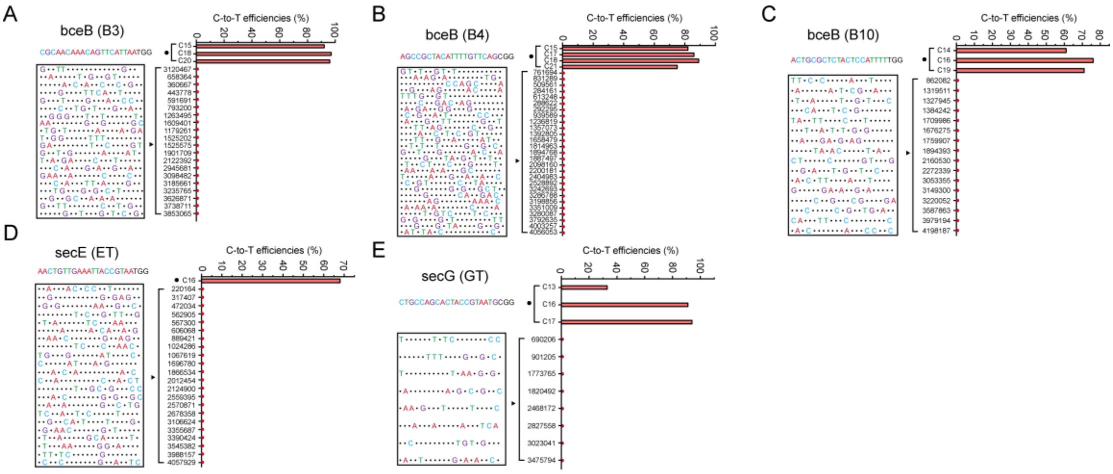

Supplementary Figure S5. Off-target editing activities of CRISPR-CDA-nCas9-UGI in *B. subtilis*. Off-target editing activities of CRISPR-CDA-nCas9-UGI was detected by NGS. (A-E) On- and off-target editing frequencies of five sgRNAs targeted to the B3 site (3110630-3110649, A), B4 site (3110357-3110376, B), B10 site (3109449-3109468, C), secE site (117620-117639, D), and secG site (3455137-3455156, E) with CRISPR-CDA-nCas9-UGI. On-target sites are marked with a black circle and off-target sites are marked with a black triangle. The red circles indicate that off-target editing activity is not detected. The sequences in the empty rectangle are possible off-target sites predicted by Cas-OFFinder (2).

REFERENCES

1. Lu, Z., Yang, S., Yuan, X., Shi, Y., Ouyang, L., Jiang, S., Yi, L. and Zhang, G. (2019) CRISPR-assisted multi-dimensional regulation for fine-tuning gene expression in *Bacillus subtilis*. Nucleic Acids Res., **47**, e40.
2. Bae, S., Park, J. and Kim, J.S. (2014) Cas-OFFinder: a fast and versatile algorithm that searches for potential off-target sites of Cas9 RNA-guided endonucleases. Bioinformatics, **30**, 1473-1475.
